# Overweight/obesity-related transcriptomic signature as a correlate of clinical outcome, immune microenvironment, and treatment response in hepatocellular carcinoma

**DOI:** 10.3389/fendo.2022.1061091

**Published:** 2023-01-12

**Authors:** Ning-Ning Feng, Xi-Yue Du, Yue-Shan Zhang, Zhi-Kai Jiao, Xiao-Hui Wu, Bao-Ming Yang

**Affiliations:** ^1^ Department of Hepatobiliary Surgery, Fourth Hospital of Hebei Medical University, Shijiazhuang, Hebei, China; ^2^ Department of Radiotherapy, Hengshui People’s Hospital, Hengshui, Hebei, China

**Keywords:** hepatocellular carcinoma, overweight, machine learning, signature, genomic alteration, immune microenvironment, sorafenib, TACE

## Abstract

**Backgrounds:**

The pandemic of overweight and obesity (quantified by body mass index (BMI) ≥ 25) has rapidly raised the patient number of non-alcoholic fatty hepatocellular carcinoma (HCC), and several clinical trials have shown that BMI is associated with the prognosis of HCC. However, whether overweight/obesity is an independent prognostic factor is arguable, and the role of overweight/obesity-related metabolisms in the progression of HCC is scarcely known.

**Materials and methods:**

In the present study, clinical information, mRNA expression profile, and genomic data were downloaded from The Cancer Genome Atlas (TCGA) as a training cohort (TCGA-HCC) for the identification of overweight/obesity-related transcriptome. Machine learning and the Cox regression analysis were conducted for the construction of the overweight/obesity-associated gene (OAG) signature. The Kaplan–Meier curve, receiver operating characteristic (ROC) curve, and the Cox regression analysis were performed to assess the prognostic value of the OAG signature, which was further validated in two independent retrospective cohorts from the International Cancer Genome Consortium (ICGC) and Gene Expression Omnibus (GEO). Subsequently, functional enrichment, genomic profiling, and tumor microenvironment (TME) evaluation were utilized to characterize biological activities associated with the OAG signature. GSE109211 and GSE104580 were retrieved to evaluate the underlying response of sorafenib and transcatheter arterial chemoembolization (TACE) treatment, respectively. The Genomics of Drug Sensitivity in Cancer (GDSC) database was employed for the evaluation of chemotherapeutic response.

**Results:**

Overweight/obesity-associated transcriptome was mainly involved in metabolic processes and noticeably and markedly correlated with prognosis and TME of HCC. Afterward, a novel established OAG signature (including 17 genes, namely, *GAGE2D*, *PDE6A*, *GABRR1*, *DCAF8L1*, *DPYSL4*, *SLC6A3*, *MMP3*, *RIBC2*, *KCNH2*, *HTRA3*, *PDX1*, *ATHL1*, *PRTG*, *SHC4*, *C21orf29*, *SMIM32*, and *C1orf133*) divided patients into high and low OAG score groups with distinct prognosis (median overall survival (OS): 24.87 *vs.* 83.51 months, p < 0.0001), and the values of area under ROC curve (AUC) in predicting 1-, 2-, 3-, and 4-year OS were 0.81, 0.80, 0.83, and 0.85, respectively. Moreover, the OAG score was independent of clinical features and also exhibited a good ability for prognosis prediction in the ICGC-LIHC-JP cohort and GSE54236 dataset. Expectedly, the OAG score was also highly correlated with metabolic processes, especially oxidative-related signaling pathways. Furthermore, abundant enrichment of chemokines, receptors, MHC molecules, and other immunomodulators as well as PD-L1/PD-1 expression among patients with high OAG scores indicated that they might have better responses to immunotherapy. However, probably exclusion of T cells from infiltrating tumors resulting in lower infiltration of effective T cells would restrict immunotherapeutic effects. In addition, the OAG score was significantly associated with the response of sorafenib and TACE treatment.

**Conclusions:**

Overall, this study comprehensively disclosed the relationship between BMI-guided transcriptome and HCC. Moreover, the OAG signature had the potential clinical applications in the future to promote clinical management and precision medicine of HCC.

## 1 Introduction

Hepatocellular carcinoma (HCC), accounting for 75%–80% of primary liver cancer, is the seventh most common cancer and occupies nearly 8.0% of all cancer-related deaths, with more than 0.9 million new cases and 0.8 million deaths worldwide (1). Currently, surgical resection and liver transplantation remain the most effective therapy for HCC patients, but most patients with advanced diseases are not suitable for surgeries (2). Despite receiving surgical treatments, 5-year overall survival (OS) rate of HCC is still poor, and relapse and metastasis rates are quite high (3). With the rapid development of sequencing technologies, comprehensive analysis of molecular characterizations offers novel insights into HCC carcinogenesis and reveals exogenous/endogenous factors potentially influencing HCC progression (4). More importantly, molecular subtyping could divide patients into different HCC subclasses with distinct prognoses, molecular features, and treatment responses altogether, which would help promote the clinical management of HCC patients and select suitable treatment regimens.

So far, HCC has been documented as a cancer type presenting a highly close relationship between tumors and environmental agents. In addition to genetic predisposition, etiological risk factors of chronic hepatitis B/C virus (HBV/HCV) infection, alcohol, tobacco smoking, obesity, contaminants/toxins, and diabetes are frequently reported to induce tumorigenesis of HCC (5). Generally, HBV/HCV-induced HCC originates from chronic liver damage, and HBV/HCV-encoded proteins could alter host transcriptome, progressively stimulating HCC cell proliferation, angiogenesis, invasion, metastasis, and reprogramming cell metabolism (6). Noticeably, alcohol consumption or abuse can greatly increase the risk for HCC, irrespective of whether concomitant HBV/HCV infection or not (7). Moreover, alcohol-related HCC patients have a worse prognosis when compared with those with non-alcoholic HCC (8). Molecular characterizations of alcohol-related HCC subtype have been intensely looked into, and some alcohol-related molecular features may serve as potential diagnostic/prognostic biomarkers or molecular targets, especially alcohol-associated metabolites (9) and alcohol metabolism-associated genes/enzymes (10) highly correlated with HCC morbidity and/or mortality. Undoubtedly, cigarette smoking is associated with a high risk of HCC; as acknowledged, smoke/nicotine exposure can aggravate HCC inflammation, suppress the anti-tumor effect of T cells, and stimulate cancer stem cell epithelial-to-mesenchymal transition, and smoke and other risk factors positively interact in the development and progression of HCC (11). A population-based study further displays that the OS time of HCC patients varies as a consequence of distinct etiological risk factors because these etiological risk factors could determine a unique molecular profile (12). Due to the epidemic of overweight/obesity over past decades, excess body weight has emerged as a closely relevant risk factor for HCC, and body mass index (BMI) is found to be positively correlated with the mortality rate of liver cancer in both men and women (13). In addition to hyperlipidemia/hypertension, metabolic syndrome, and diabetes, overweight/obesity or higher BMI becomes one of the major risk factors for non-alcoholic fatty liver disease (NAFLD), which is highly correlated with the development of HCC, particularly within those having NAFLD-related cirrhosis and fibrosis (14). Moreover, approximately 20%–30% of NAFLD-related HCC cases develop into HCC in the absence of cirrhosis and fibrosis, and NAFLD is a leading cause of HCC in the absence of cirrhosis and fibrosis (15). Overall, increasing pieces of evidence have disclosed the relationship between overweight/obesity (or high BMI) and tumor progression; however, comprehensive molecular characterizations related to overweight/obesity (or high BMI) in HCC remain to be fully elucidated.

The present study is the first time to reveal that overweight/obesity-related transcriptomic features could distinguish HCC patients with distinct prognoses, biological metabolism, and the immune microenvironment. Based on this overweight/obesity-related transcriptome, a novel overweight/obesity-associated gene (OAG) signature together with a scoring system was subsequently constructed. From a new perspective, the underlying signaling pathways, genomic alterations, and tumor microenvironment were deeply investigated in HCC. Intriguingly, the OAG score was also found to be closely correlated with sorafenib and transcatheter arterial chemoembolization (TACE) treatment responses; furthermore, the OAG score was also of guiding significance to evaluate chemotherapy response.

## 2 Materials and methods

### 2.1 Data collection and preprocessing

In the present study, clinical information, mRNA expression data, and genomic data of 360 HCC patient samples (cases without complete information were excluded) were retrieved from The Cancer Genome Atlas (TCGA) *via* the cBioPortal (https://www.cbioportal.org/), regarded as the training cohort. As mentioned earlier, alcohol consumption might aggravate the development and progression of HCC; thus, patients with a risk history of alcohol consumption were excluded. The remaining 199 patient samples were collected to explore the relationship between overweight/obesity and the OS of HCC patients and identify the differentially expressed genes (DEGs) between patients presenting with overweight/obesity or not. In addition, a total of 232 HCC patient samples from the International Cancer Genome Consortium (ICGC; https://dcc.icgc.org/projects/LIRI-JP, namely, ICGC-LIHC-JP) and 72 patient samples selected from the GSE54236 dataset in the Gene Expression Omnibus (GEO; https://www.ncbi.nlm.nih.gov/geo/), respectively, were downloaded as two independent validation cohorts.

### 2.2 Overweight/obesity-associated transcriptome and unsupervised hierarchical clustering analysis

Of the selected 199 HCC patients in the training cohort, 91 and 108 cases had BMI over 25 and below 25, respectively. The mRNA expression data, with the format of fragments per kilobase million (FPKM), were initially normalized by log_2_ (FPKM + 0.001) and then utilized for the DEG analysis (p < 0.05, |log_1.5_ (fold change)| > 1) between patient samples with BMI over 25 and below 25, by using the package “DeSeq2”. The result of the DEG analysis was exhibited *via* the volcano plot by using the package “ggplot2”. Based on the overweight/obesity-derived DEGs, which were also defined as the integrated overweight/obesity-associated transcriptome, unsupervised hierarchical clustering separated this part of HCC patients into different clusters by using the package “Fastcluster”. The Kaplan–Meier curve analysis was conducted to compare the OS of different clusters by using the package “survival”. Similarly, in the whole TCGA-HCC cohort, unsupervised hierarchical clustering by a foundation of overweight/obesity-associated transcriptome also distinguished two clusters (clusters 1 and 2), and the principal component analysis (PCA) was conducted to display the discrepancy of these two clusters by using the package “ggbiplot”.

### 2.3 Functional enrichment analysis

Based on the DEGs between different clusters, Gene Ontology (GO; http://geneontology.org/) and Kyoto Encyclopedia of Genes and Genomes (KEGG; https://www.kegg.jp/) pathway enrichment analyses (16, 17) were performed by using the package “clusterProfiler” to exhibit the biological activities underlying overweight/obesity-associated transcriptome.

### 2.4 Tumor microenvironment evaluation

Additionally, tumor purity, ESTIMATE, and TIDE scores were employed to evaluate the tumor microenvironment (TME) of these two clusters (18, 19). Based on the bulk mRNA expression data, a total of 122 immune-related modulators, including chemokines, MHC molecules, receptors, and other immunomodulators, were retrieved to estimate the immunological characteristics (20). The expression of 122 immunomodulators was exhibited by using the package “pheatmap”. The cancer immunity cycle, containing seven steps and reflecting the anti-cancer immune response, was used to determine the activities of anti-cancer immunity (21). The single-sample gene set enrichment analysis (ssGSEA) was conducted to characterize the activity of each step (22). Finally, multiple kinds of immune checkpoint gene expression profiles were investigated (23).

### 2.5 Machine learning for the construction of a novel OAG signature

Initially, mRNA expression data of HCC tumor and normal samples were downloaded from the data portal of UCSC xena (https://xenabrowser.net/datapages/) to identify HCC-associated DEGs. Next, the overlapping gene set between overweight/obesity-associated DEGs and HCC-associated DEGs was collected, which was visualized in a Venn plot by using the package “eulerr”. The overlapping genes were then enrolled into the univariate Cox regression analysis by using the package “rms” to screen out the OS-related genes. Subsequently, the random forest (RF) algorithm was used to select the representative genes (normalized variable importance measure index > 0.40) by using the package “randomSurvivalForest”. Based on the expression of representative genes, the least absolute shrinkage and selection operator (LASSO) Cox regression analysis was conducted to construct a novel OAG signature by using the package “glmnet”; correspondingly, the OAG score of each sample was calculated by the following formula:


$$OAGs\;score\;=\;\sum_{x=1}^{n}OAG_{x}*Coef_{x}$$

where *n*, *OAG_x_*, and *Coef_x_* represent the number of OAGs included in the signature, OAG expression level, and coefficient value, respectively.

In TCGA-HCC cohort, patients were assigned to the high and low OAG score groups according to the median OAG score as the cutoff value. The Kaplan–Meier curve analysis was conducted to compare the OS between these two groups. The receiver operating characteristic (ROC) curve analysis, quantified by the value of area under the ROC curve (AUC), was utilized to evaluate the performance of the OAG score in prognosis prediction by using the package “rms”. In addition, the Kaplan–Meier curve and ROC curve analysis were also conducted in the ICGC-LIHC-JP cohort and GSE54236 dataset to validate the robustness of the OAG score in prognosis prediction. Furthermore, we compared the predictive accuracy of the OAG signature with other risk signatures, including immune- (24), mitochondrial- (25), energy metabolism- (26), ferroptosis- (27), cuprotosis- (28), and TGF-β-related (29) signatures. The univariate and multivariate Cox regression analyses were conducted to recognize whether the OAG score was an independent prognostic factor.

### 2.6 Single OAG analysis and immunohistochemistry staining

Regarding the role of single OAG expression in HCC, the heatmap plot demonstrated the detailed information of each signature-related OAG expression and corresponding clinical features in samples from TCGA-HCC cohort. Moreover, Pearson’s correlation analysis was conducted to investigate the correlation of each OAG expression. Underlying a single OAG expression, the Kaplan–Meier curve analysis was conducted to exhibit the prognostic significance of OAGs; meanwhile, the ROC curve analysis was also performed for each OAG. Eventually, OAG protein expression was analyzed by immunohistochemistry (IHC) staining using the available HCC tumor macro-array staining from the Human Protein Atlas (https://www.proteinatlas.org/). Collectively, 10–12 HCC samples were analyzed for the expression of DPYSL4, MMP3, HTRA3, PDX1, C21orf29, ATHL1, PDE6A, DCAF8L1, SLC6A3, and RIBC2 proteins, while there was no information of IHC staining for the expression of GABRR1, GAGE2D, KCNH2, PRTG, SHC4, and SMIM32 proteins. Also, there was no IHC staining information on *C1orf133*, which was a kind of non-coding RNA (ncRNA).

### 2.7 Molecular characterizations associated with OAG score

Based on the DEGs between the high and low OAG score groups, GO and KEGG pathway enrichment analyses were initially conducted to identify the critical biological activities/pathways associated with the OAG score. First, the gene set enrichment analysis (GSEA) was performed by using the package “GSVA”, and Hallmark gene sets were obtained for GSEA (https://www.gsea-msigdb.org/gsea/msigdb/genesets.jsp?collection=H). Second, genomic alteration data in TCGA-HCC cohort was employed to visualize the discrepancy between the high and low OAG score groups by using the package “maftools”; meanwhile, the CoMEt algorithm was utilized to investigate the co-occurrence and mutually exclusive alterations (30). The specific alteration sites of the prevalent genes were exhibited *via* the lollipop plot. Same as described before, TME characteristics associated with OAGs were lastly investigated by the following indexes: tumor purity, ESTIMATE, TIDE, and the infiltration of 22 immune cells. Immunological characteristics of immunomodulators, cancer immunity cycle, and immune checkpoint gene expression associated with the OAG score were also compared between the high and low OAG score groups.

### 2.8 Estimate of treatment responses by sorafenib, TACE, and chemical drugs

As known, sorafenib, TACE, and chemotherapeutic treatments are usually selected for HCC patients. GSE109211 dataset (31), composed of 21 responders and 46 non-responders (https://www.ncbi.nlm.nih.gov/geo/query/acc.cgi?acc=GSE109211) when receiving sorafenib treatment, was downloaded to explore whether the OAG score or OAG expression was correlated with sorafenib treatment response in HCC. Subsequently, the GSE104580 dataset (https://www.ncbi.nlm.nih.gov/geo/query/acc.cgi?acc=GSE104580) of 147 HCC patients treated with TACE treatment, including 81 responders and 66 non-responders, respectively, was retrieved to investigate the correlation between the OAG score or OAG expression and response to TACE treatment. In addition, the Genomics of Drug Sensitivity in Cancer (GDSC; https://www.cancerrxgene.org/) database of pharmacogenomic data was downloaded to calculate the half-maximal inhibitory concentration (IC50 value), which was used for chemotherapeutic response prediction. In the present study, cisplatin, 5-fluorouracil (5-FU), paclitaxel, vinblastine, and other commonly used chemical drugs were evaluated.

### 2.9 Statistical analysis

**Figure 1:**
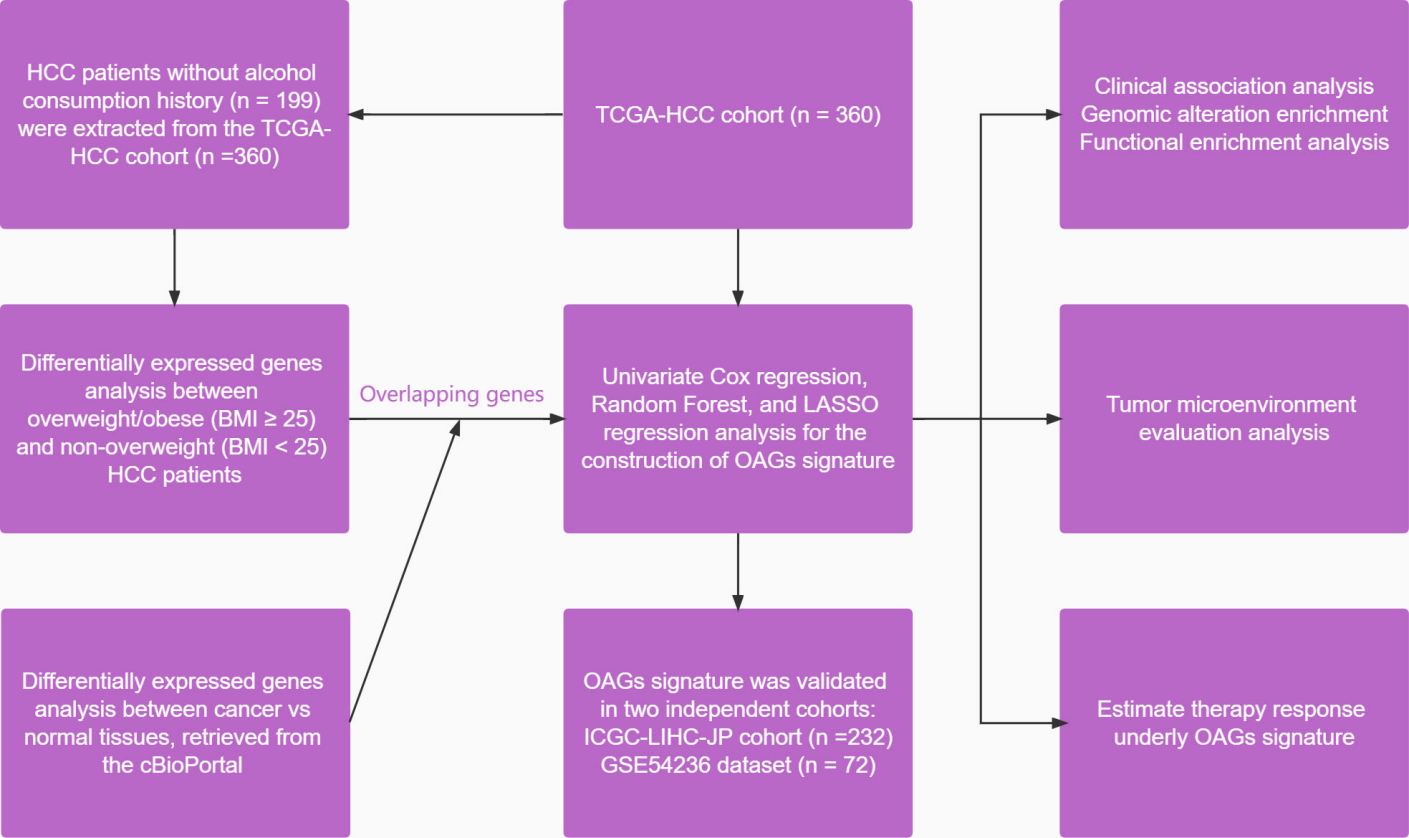
A flowchart of study design in the present study.

All statistical analyses were conducted in the present study *via* the R software (version 4.1.1). Fisher’s exact test and Student’s t-test were used for comparisons of categorical variables and continuous variables. Moreover, the Wilcoxon test and Kruskal–Wallis test were applied for comparisons between two and multiple comparisons. The Kaplan–Meier curve analysis was conducted using the log-rank test. The univariate and multivariate Cox regression analyses were used to disclose the factors associated with survival. The correlation between variables was calculated by using Pearson’s coefficient. The significant difference was considered with at least p < 0.05. The overall study design is shown in **Figure 1**.

## 3 Results

### 3.1 Identification of overweight/obesity-associated transcriptome among patients not using alcohol

As previously described, alcohol consumption significantly increased the risk of HCC; correspondingly, HCC-related symptoms aggravated gradually. In line with previous findings, we did not observe any significant difference in OS between HCC patients with distinct BMI in the whole TCGA-HCC cohort (**Table 1**; **Figure S1A**). As those patients with alcohol consumption were excluded (**Table S1**), intriguingly, remaining HCC patients with overweight/obesity (BMI ≥ 25) tended to have a worse OS (median OS, 51.25 months *vs.* unreached, p = 0.34, **Figure S1B**). Between HCC patients with BMI ≥25 and <25, a total of 882 DEGs were identified (p < 0.05, |log_1.5_ (fold change)| > 1, **Figure S1C**), and these DEGs were mainly enriched in the biological activities of metabolic processes, oxygen transport, stem cell proliferation, and WNT protein binding (**Figure S1D**). Based on the expression of 882 DEGs, unsupervised hierarchical clustering analysis (**Figure S1E**) identified two subgroups with distinct OS (median OS, 51.25 months *vs.* unreached, p = 0.0019, **Figure S1F**) among HCC patients without alcohol consumption.

**Table 1:** Patient characteristics in TCGA-HCC cohort.

Features		Number
Total		360
Age	Median (range)	61 [16, 90]
Gender	Male	242
	Female	118
Alcohol use	Used	161
	Other	199
Body mass index	<25	173
	≥25	154
Vascular invasion	Macro	16
	Micro	89
	None	202
Histological grading	G1	54
	G2	171
	G3	118
	G4	12
T stage	T1	177
	T2	90
	T3	77
	T4	13
	NA	3
N stage	N0	247
	N1	3
M stage	M0	260
	M1	3
Clinical stage	I	169
	II	84
	III	82
	IV	4
HBV/HCV status	HBV positive	94
	HCV positive	211
	HBV and HCV positive	7
	HBV and HCV negative	48

HBV/HCV, hepatitis B/C virus.

### 3.2 Overweight/obesity-associated transcriptome and functional annotation

**Figure 2:**
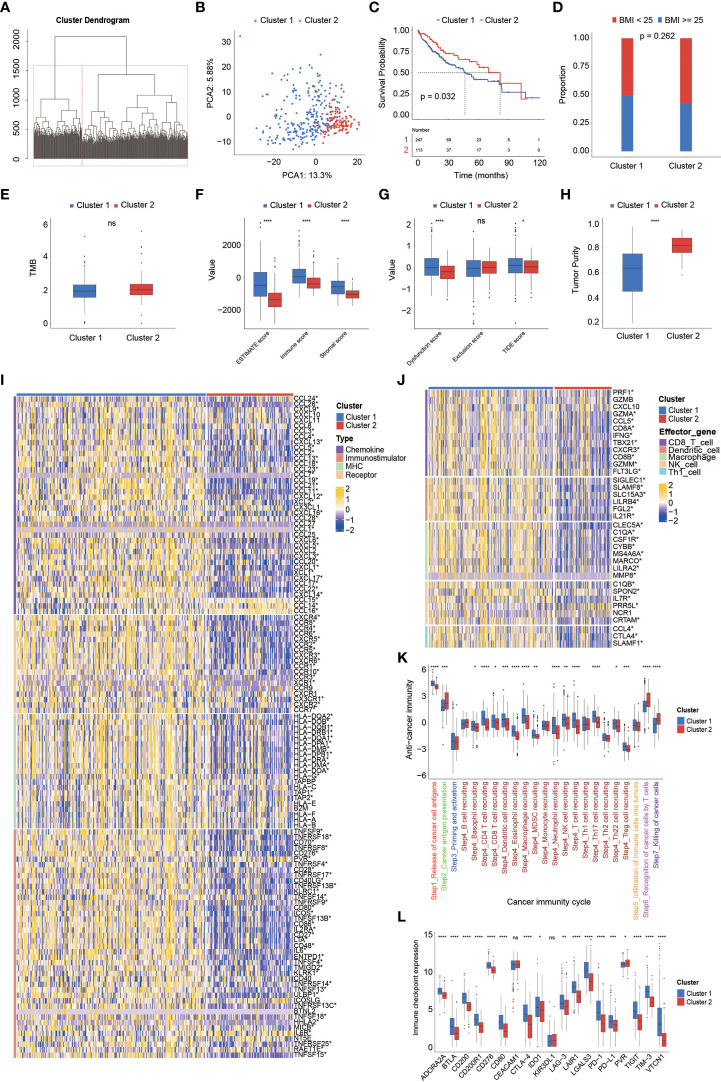
The overweight-associated transcriptome highly correlated with prognosis, immune characteristics, and anti-cancer immunity in TCGA-HCC cohort. **(A)** Unsupervised hierarchical clustering by foundation of overweight-associated transcriptome in TCGA-HCC cohort. **(B)** Principal component analysis for two clusters. **(C)** Kaplan–Meier curve analysis for two clusters. **(D)** Proportional analysis of patients with overweight/obesity between these two clusters. **(E–H)** Comparison of TMB level **(E)**, ESTIMATE score **(F)**, TIDE score **(G)**, and tumor purity **(H)** between clusters 1 and 2. **(I)** Differences in the expression of immunomodulators (chemokines, receptors, MHC molecules, and other immunomodulators) between clusters 1 and 2. **(J)** Evaluation of effector gene expression of tumor-infiltrating immune cells. **(K)** Comparison of cancer immunity cycles between clusters 1 and 2. **(L)** Comparison of immune inhibitory checkpoint expression between clusters 1 and 2. TMB, tumor mutational burden.

When the whole TCGA-HCC cohort was considered as a training cohort, the overweight/obesity-associated transcriptome also differentiated two clusters (**Figure 2A**) with significantly different OS (median OS: cluster 1 *vs.* cluster 2, 46.75 months *vs.* 81.67 months, p = 0.032, **Figures 2B, C**), and there was a higher proportion of patients with overweight/obesity in cluster 1 (p = 0.262, **Figure 2D**). Functional enrichment revealed that overweight/obesity-associated transcriptome was highly correlated with fatty acid metabolism, cytochrome P450-mediated metabolism, oxidative signaling pathways, and multiple cancer-related metabolisms (**Figure S2**). Furthermore, no significant difference in tumor mutational burden (TMB) was observed between the two clusters (**Figure 2E**), but it was noticeable that cluster 1 had higher ESTIMATE and TIDE scores but lower tumor purity (**Figures 2F–H**). In addition, a large number of chemokines, receptors, MHC molecules, and immunomodulators (**Figure 2I**) as well as the effector genes of CD8+ T cells, dendritic cells, macrophages, NK cells, and Th1 cells were upregulated in cluster 1 (**Figure 2J**). Correspondingly, activities of Steps 1 (release of cancer cell antigens) and 4 (trafficking of immune cells tumors) were upregulated in cluster 1; however, activities of Steps 2 (cancer antigen presentation), 6 (recognition of cancer cells by T cells), and 7 (killing of cancer cells) were downregulated (**Figure 2K**), while the expression of most immune checkpoint genes, including *PD-L1*, *PD-1*, *CTLA-4*, *LAG-3*, *TIGIT*, *TIM-3*, *CD80*, *CD200*, and *CD276*, was markedly upregulated, but only the expression of *PVR* was downregulated in cluster 1 (**Figure 2L**).

### 3.3 A novel OAG score as correlate of prognosis of HCC patients

**Figure 3:**
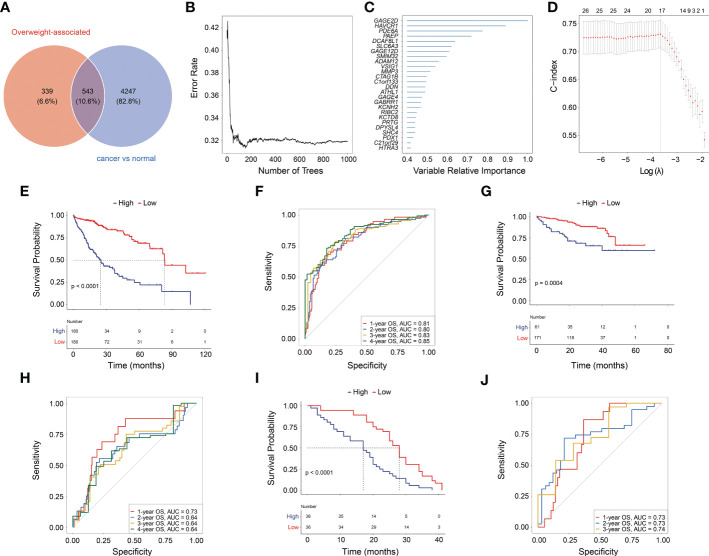
Machine learning for construction of overweight-associated gene (OAG) signature. **(A)** Overlapping of overweight-associated genes and differentially expressed genes between cancer and normal samples. **(B)** The random forest algorithm for identification of key genes correlated with overall survival (OS) in HCC. **(C)** The evaluation of the importance of selected OAGs. **(D)** The least absolute shrinkage and selection operator (LASSO) Cox regression analysis for determining an OAG signature and corresponding scoring system. **(E)** Kaplan–Meier curve analysis between high and low OAG score in TCGA-HCC cohort. **(F)** The receiver operating characteristic (ROC) curve analysis of OAG signature in prognosis prediction in TCGA-HCC cohort. **(G)** Kaplan–Meier curve analysis between high and low OAG score in ICGC-LIHC-JP cohort. **(H)** The ROC curve analysis of OAG signature in prognosis prediction in ICGC-LIHC-JP cohort. **(I)** Kaplan–Meier curve analysis between high and low OAG scores in GSE54236 dataset. **(J)** The ROC curve analysis of OAG signature in prognosis prediction in GSE54236 dataset. HCC, hepatocellular carcinoma.

Given overweight/obesity-associated metabolic transcriptome, RF algorithm and LASSO Cox regression analysis were conducted to construct an OAG signature. Initially, it was discovered that 543 of 882 OAGs were differentially expressed between normal and tumor samples (**Figure 3A**; **Table S2**), among which the expression of 262 OAGs was significantly correlated with OS of HCC patients in TCGA-HCC cohort (**Table S3**). Next, the RF algorithm screened out the most representative 26 OAGs (**Figures 3B, C**). After the over-fitting by the LASSO Cox regression analysis was minimized, a novel signature consisting of 17 OAGs together with an OAG signature scoring system was constructed (**Figure 3D**; **Table 2**). According to the median cutoff value, the OAG score separated TCGA-HCC cohort population into two distant groups, termed the high and low OAG score groups. Comparatively, the high OAG score group had quite worse OS (median OS, 24.87 *vs.* 83.51 months, p < 0.0001, **Figure 3E**). Noticeably, the AUC values of the OAG score in predicting 1-, 2-, 3-, and 4-year OS were 0.81, 0.80, 0.83, and 0.85, respectively (**Figure 3F**), suggesting that a novel OAG signature performed well in prognosis prediction. Subsequently, the OAG signature was further verified in two independent cohorts, ICGC-LIHC-JP cohort (**Table S4**) and GSE54236 dataset (**Table S5**), and indeed, it was observed that the OAG score was negatively correlated with OS (median OS in ICGC-LIHC-JP cohort: unreached *vs.* unreached, p = 0.0004; GSE54236 dataset, 16.98 *vs.* 28.01 months, p < 0.0001, **Figures 3G–J**). Within these two independent validation cohorts, almost all AUC values of the OAG score in predicting OS were relatively high, confirming that the OAG signature is reliable and robust in prognosis prediction. When being compared with already reported prognostic signatures, such as immune, mitochondria, energy metabolism, ferroptosis, cuprotosis, and TGF-β related signatures, the OAG signature outperformed in prognosis prediction (**Figure S3**). For HCC patients without alcohol consumption, the OAG signature seemed to perform better in prognosis prediction (**Figure S4**), and the high OAG score group had a higher proportion of patients with overweight/obesity (51.01% *vs.* 41.10%, p = 0.215) when compared with the low OAG score group.

**Table 2:** A total of 17 genes included in overweight-associated genes signature.

Gene name	HR	95% CI	Coefficient
*ATHL1*	0.5179	0.3643−0.7362	−0.1429
*SMIM32*	0.4909	0.3438−0.7009	−0.1096
*PRTG*	0.5421	0.3770−0.7794	−0.0026
*SHC4*	0.5573	0.3888−0.7988	−0.0411
*C21orf29*	0.5339	0.3666−0.7775	−0.0306
*C1orf133*	0.4811	0.3293−0.7026	−0.1294
*MMP3*	2.3626	1.6101−3.4668	0.0305
*GABRR1*	2.1684	1.5228−3.0877	0.1039
*GAGE2D*	2.7373	1.8823−3.9806	0.1021
*DPYSL4*	2.2922	1.3895−3.7813	0.0307
*SLC6A3*	1.7895	1.2593−2.5429	0.0086
*RIBC2*	1.6269	1.1503−2.3011	0.0086
*DCAF8L1*	1.6812	1.1560−2.4449	0.0157
*PDE6A*	1.5970	1.0556−2.4161	0.1873
*KCNH2*	1.5493	1.0335−2.3225	0.0740
*HTRA3*	1.6280	1.1363−2.3324	0.0641
*PDX1*	1.4462	1.005−2.0814	0.0439

### 3.4 Prognostic significance and contribution of OAGs

**Figure 4:**
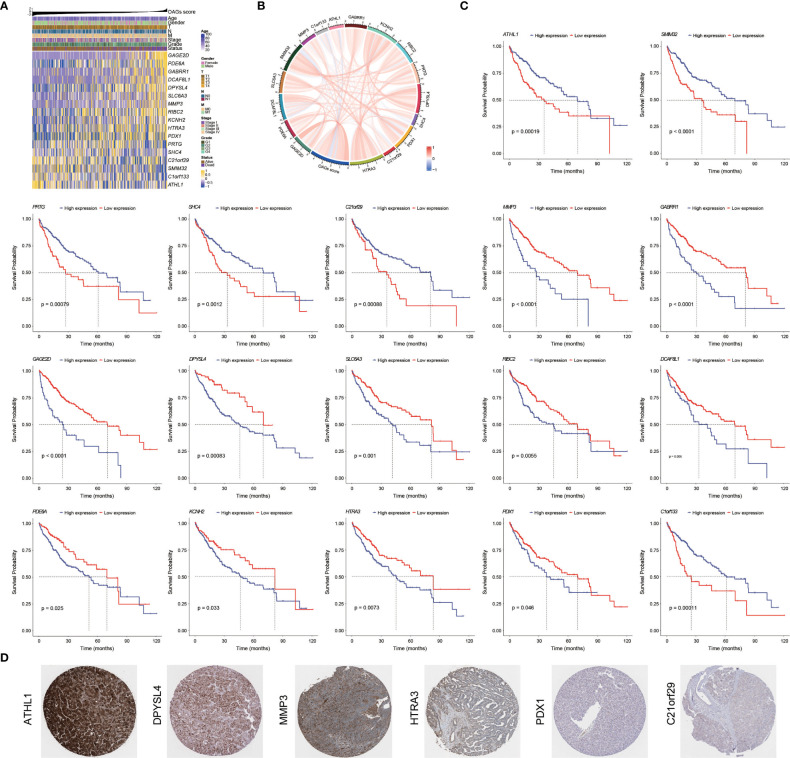
Prognostic significance and contribution of overweight-associated genes (OAGs) involved in the signature. **(A)** The heatmap for OAG expression profiling. **(B)** The correlation between OAG expression and OAG score. **(C)** Kaplan–Meier curve analysis based on the expression of single OAG. **(D)** The immunohistochemistry staining of OAG protein expression in TCGA-HCC samples.

Overall, there were 11 and 6 OAGs serving as OS-related risk factors and protective factors, respectively (**Figure 4A**). In TCGA-HCC cohort, the expression of *GAGE2D*, *PDE6A*, *GABRR1*, *DCAF8L1*, *DPYSL4*, *SLC6A3*, *MMP3*, *RIBC2*, *KCNH2*, *HTRA3*, and *PDX1* remarkably increased in the high OAG score group, and the expression of each OAG was positively associated with the OAG score, whereas the expression of *ATHL1*, *PRTG*, *SHC4*, *C21orf29*, *SMIM32*, and *C1orf133* (ncRNA) elevated in the low OAG score group, and their expression was negatively associated with the OAG score (**Figures 4A, B**). As expected, the overexpression of 11 risk-related OAGs indicated poorer OS, but the overexpression of six protective-related OAGs was correlated with prolonged OS in HCC (p < 0.05, **Figure 4C**). It was noteworthy that the AUC values of *ATHL1*, *GAGE2D*, and *RIBC* in predicting 1-, 2-, 3-, and 4-year OS were all beyond 0.60, although the predictive ability of single OAG was inferior to that of the OAG signature (**Figure S5**). In addition, it was observed that 11/11, 11/11, 4/12, 2/12, 1/11, and 12/12 HCC samples expressed DPYSL4, MMP3, HTRA3, PDX1, C21orf29, and ATHL1 proteins in the cytoplasm/membrane, respectively (**Figure 4D**), but the IHC staining of PDE6A (0/11), DCAF8L1 (0/11), SLC6A3 (0/11), and RIBC2 (0/10) was negative. In contrast, in the Human Protein Atlas (HPA) database, there was no information on the IHC staining of GABRR1, GAGE2D, KCNH2, PRTG, SHC4, and SMIM32. Moreover, *C1orf133* belonged to ncRNA.

### 3.5 Association between independent OAG score and clinical features

A combination of univariate and multivariate Cox regression analyses revealed that the OAG score was a robust prognostic factor (HR = 5.20, 95% CI, 2.55–10.60, p < 0.0001), which was independent of clinical features in TCGA-HCC cohort (**Table 3**). More importantly, the OAG score was a better predictor of OS for HCC patients than the baseline clinical characteristics (**Figure S6**). Regarding the relationship between an independent OAG score and clinical features, the high OAG score group had more HCC patients with higher alpha-fetoprotein (AFP) levels (p < 0.01), T stages (p < 0.01), clinical stages (p < 0.05), grades (p < 0.05), and macro- or micro-vascular invasions but a lower proportion of HBV-infected HCC patients (p < 0.001, **Figure S7**). Correspondingly, the OAG score was positively correlated with AFP level (p < 0.001), T stage (p < 0.001), clinical stage (p < 0.001), high grade (p < 0.05), and vascular invasion, and HBV-infected HCC patients had the lowest OAG score (p < 0.001, **Figure S8**). Owing to a limited number of patients with lymph node metastasis or distant metastasis, there was no discrepancy in the OAG score between N0 and N1+ stage groups or M0 and M1 stage groups. As for distinct HCC subtypes that were separated by these baseline clinical features, the OAG score still exhibited excellent performance in prognosis prediction, and the high OAG score group always had an inferior OS (p < 0.05, **Figure S9**). Moreover, stratification analysis demonstrated that the OAG score could potentially predict prognosis for early-stage HCC patients.

**Table 3:** Univariate and multivariate Cox regression analyses for OAG score and clinical features in TCGA-HCC cohort.

Variable	Univariate			Multivariate		
	HR	95% CI	p-Value	HR	95% CI	p-Value
OAG score						
High/low	4.60	3.09–6.83	<0.01^**^	4.26	2.32–7.81	<0.01^**^
Age						
≥61/<61	1.28	0.90–1.80	0.17	2.13	1.18–3.85	<0.05^*^
Gender						
Male/female	0.81	0.57–1.15	0.23	1.59	0.83–3.03	0.16
Body mass index						
≥ 25/<25	0.80	0.56–1.17	0.25	0.88	0.50–1.53	0.64
Alcohol use						
Yes/no	1.08	0.75–1.57	0.68	0.53	0.25–1.14	0.10
Vascular invasion						
Yes/no	1.34	0.89–2.03	0.16	1.12	0.63–1.99	0.70
Grade						
High/low	1.11	0.78–1.60	0.56	1.73	0.98–3.06	0.06
T stage						
High/low	2.47	1.73–3.52	<0.01^**^	1.05	0.12–8.83	0.97
N stage						
N1/N0	1.19	0.17–8.60	0.86	1.17	0.39–5.18	0.32
M stage						
M1/M0	4.06	1.27–12.9	0.02^*^	2.63	0.71–9.69	0.15
Clinical stage						
High/low	2.38	1.64–3.45	<0.01^**^	1.80	0.22–14.6	0.58
Virus status						
Positive/negative	0.52	0.35–0.76	<0.01^**^	0.67	0.35–1.31	0.25

OAG, overweight/obesity-associated gene.

*: p < 0.05; **: p < 0.01.

### 3.6 OAG score-associated tumors with different metabolic characteristics

**Figure 5:**
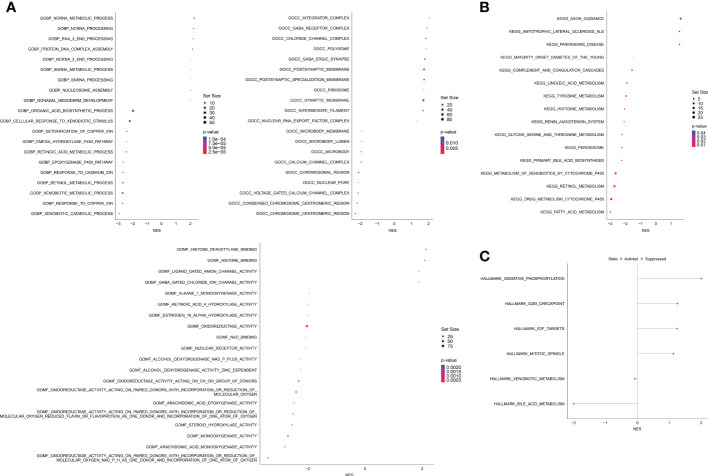
Biological enrichment analysis between high and low OAG score groups. **(A)** Gene ontology enrichment analysis. **(B)** Kyoto Encyclopedia of Genes and Genomes enrichment analysis. **(C)** Gene set enrichment analysis based on the Hallmark pathways. OAG, overweight/obesity-associated gene.

A total of 2,502 DEGs (p < 0.05, |log_1.5_ (fold change)| > 1, **Table S6**) were identified between the high and low OAG score groups. These genes were subsequently enrolled into functional enrichment analysis to evaluate the differential biological activities and signaling pathways between the high and low OAG score groups. GO and KEGG pathway enrichment analyses showed that fatty acid metabolism, cytochrome P450-mediated metabolism, amino acid metabolism, retinol metabolism, and xenobiotic metabolism were majorly involved (**Figures 5A, B**). Noticeably, GO and KEGG pathway enrichment analyses underlying a single OAG resulted in similar findings (**Table S7**). The GSEA of Hallmark pathways revealed that oxidative phosphorylation and cell cycle/DNA replication-related signaling pathways, including G2M checkpoint, E2F targets, and mitotic spindle, were significantly enriched in the high OAG score group (**Figure 5C**), whereas bile acid and xenobiotic metabolism were suppressed in the high OAG score group.

### 3.7 OAG score associated with distinct somatic genome

**Figure 6:**
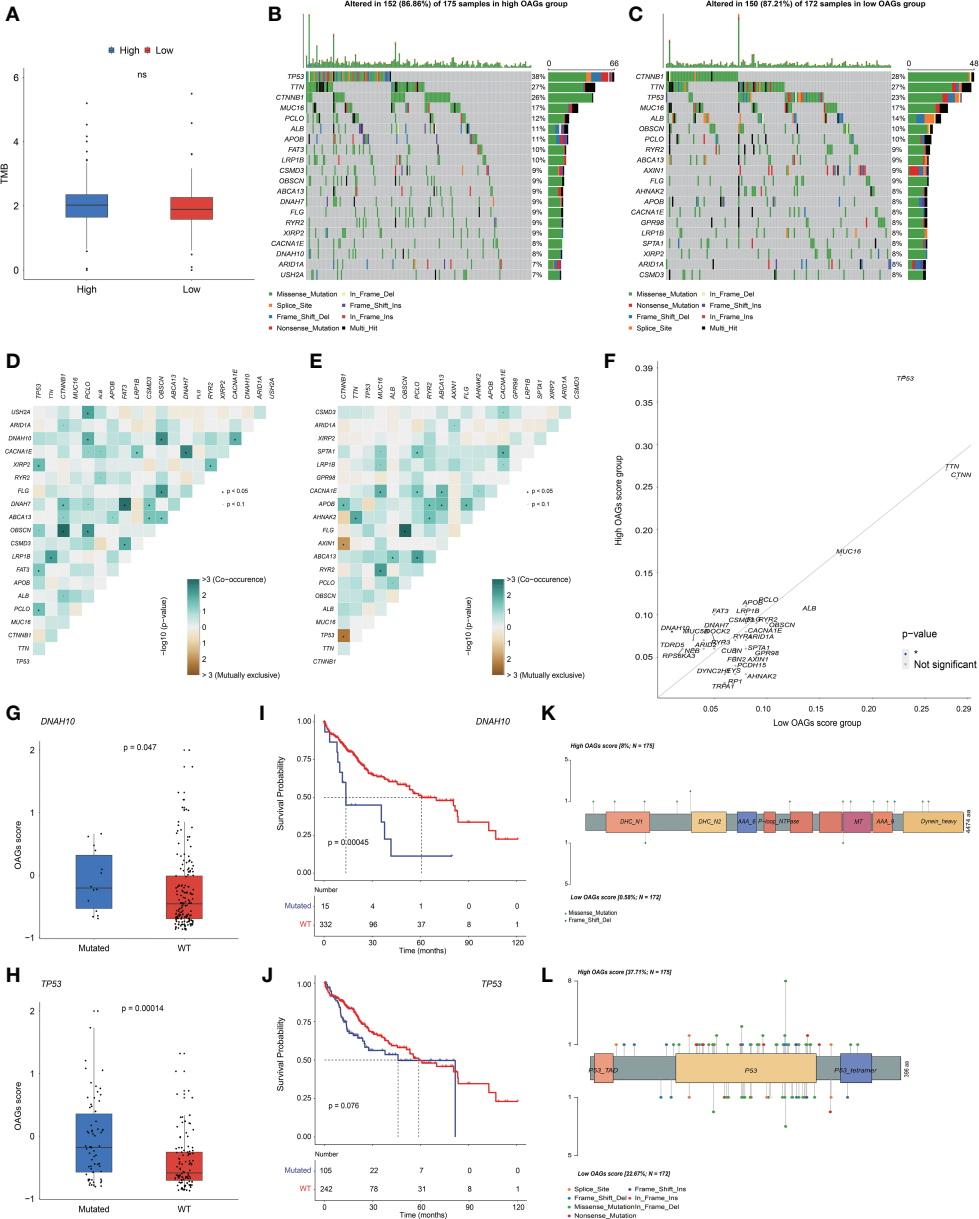
OAG score associated with distinct somatic genome. **(A)** The evaluation of TME level between high and low OAG score groups. **(B)** Oncoprint plot for genomic alterations of patients from high OAG score group. **(C)** Oncoprint plot for genomic alterations of patients from low OAG score group. **(D)** The heatmap of mutually co-occurrence and exclusive alterations of the top 20 altered genes in high OAG score group. **(E)** The heatmap of mutually co-occurrence and exclusive alterations of the top 20 altered genes in low OAG score group. **(F)** The somatic alteration enrichment analysis for high and low OAG score groups. **(G)** *DNAH10* somatic alteration associated with OAG score. **(H)** *TP53* somatic alteration associated with OAG score. **(I)** Kaplan–Meier curve analysis between patients with *DNAH10* somatic alterations or not. **(J)** Kaplan–Meier curve analysis between patients with *TP53* somatic alterations or not. **(K)** The profiling of alteration sites of *DNAH10* somatic alterations between high and low OAG score groups. **(L)** The profiling of alteration sites of *TP53* somatic alterations between high and low OAG score groups. OAG, overweight/obesity-associated gene; TME, tumor microenvironment.

Likewise, there was no significant association between the OAG score and TMB level (**Figure 6A**). Based on the whole-exome sequencing (WES) data from TCGA-HCC cohort, it was identified that 52 genes and 52 genes were altered in more than 5% of patient samples in the high and low OAG score groups, respectively (**Table S8**). Subsequently, oncoprint plots illustrated the top 20 most prevalently altered genes in the corresponding groups (**Figures 6B, C**). Collectively, most genomic alterations were missense; meanwhile, *TP53*, *TTN*, and *CTNNB1* occupied the top three positions in both groups. Based on the top 20 most frequently altered genes in the high and low OAG score groups, it was found that co-occurrence landscapes were distinct between the high and low OAG score groups (**Figures 6D, E**), and interestingly, significantly co-occurrence pairs were enriched in both groups except two special pairs (*CTNNB1-AXIN1* and *CTNNB1-TP53*) in the low OAG score group, demonstrating mutually exclusive alterations (**Figure 6E**). By further statistical analysis, it was highlighted that *TP53* (37.71% *vs.* 22.67%) and *DNAH10* (8.00% *vs.* 0.58%) were significantly more prevalent in the high OAG score group; but comparatively, none of the genes was significantly more altered in the low OAG score group instead (**Figure 6F**). Furthermore, *TP53* or *DNAH10* alterations were positively correlated with the OAG score (**Figures 6G, H**), and correspondingly, the *TP53* or *DNAH10* altered group had inferior OS indeed (**Figures 6I, J**). The in-depth investigation of specific altered locations did not recognize any difference between these two groups (**Figures 6K, L**).

### 3.8 TME characteristics associated with OAG score

**Figure 7:**
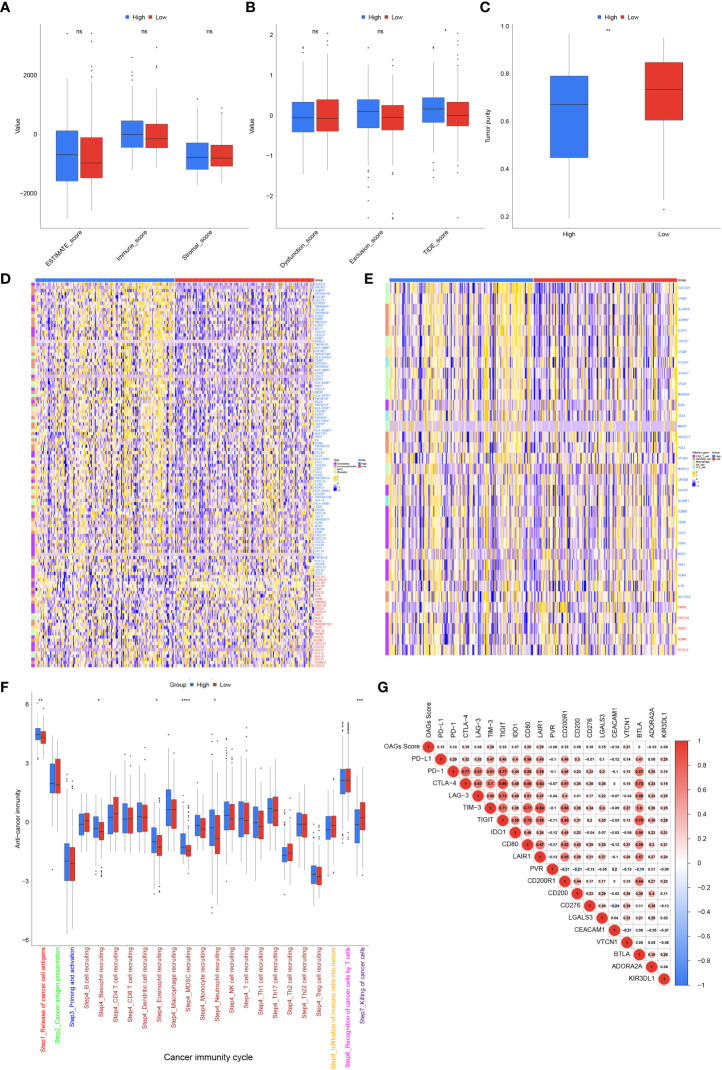
Tumor microenvironment (TME) associated with OAG score. **(A)** The stromal, immune, and ESTIMATE scores between high and low OAG score groups. **(B)** The dysfunction, exclusion, and TIDE score between high and low OAG score groups. **(C)** The evaluation of tumor purity. **(D)** Comparison of immunomodulator-related gene expression between high and low OAG score groups. **(E)** Transcriptomic profiling of effector genes of tumor-infiltrating immune cells in high and low OAG score groups. **(F)** Evaluation and comparison of anti-cancer immunity by cancer immunity cycle between high and low OAG score groups. **(G)** Correlation between OAG score and immune inhibitory checkpoint gene expression. OAG, overweight/obesity-associated gene.

Subsequently, TME was further evaluated between the high and low OAG score groups. Although there was no statistically significant difference in ESTIMATE score between these two groups, a higher OAG score indicated an increased TIDE (p < 0.05) score but lower tumor purity (p < 0.01, **Figures 7A–C**). Moreover, it was identified that indeed the expression of chemokines (*CCL7*, *CCL13*, *CCL20*, *CCL26*, *CXCL1*, *CXCL3*, *CXCL5*, and *CXCL6*), paired receptors (*CCR1*, *CCR3*, *CCR8*, *CCR10*, *CXCR2*, and *CXCR4*), and a large number of MHC molecules (*HLA-DQA*, *HLA-DOB*, *HLA-DQB1*, *HLA-DPA1*, *HLA-DMB*, *HLA-DRA*, *HLA-DMA*, *HLA-DOA*, *TAP1*, and *TAP2*) significantly elevated in the high OAG score group (**Figure 7D**). The expression of *CCL14*, *CCL15*, *CCL16*, *IL6R*, and *ICOSLG* was upregulated in the low OAG score group. Furthermore, the OAG score was also positively correlated with a majority of other immunomodulators. Notably, it was further found that there was almost no significant difference in the expression of effector genes of CD8+ T cells, NK cells, and Th1 cells, although several dendritic cell- and macrophage-associated effector genes, including *SLAMF8*, *LILRB4*, *IL21R*, *CLEC5A*, *C1QA*, *CSF1R*, *CYBB*, and *LILRA2*, were significantly upregulated in the high OAG score groups (**Figure 7E**). Correspondingly, cancer immunity cycle activity analysis revealed that the release of cancer cell antigens (Step 1) and trafficking of immune infiltrating cells to tumor cells (Step 4: basophil recruitment, eosinophil recruitment, myeloid-derived suppressor cell (MDSC) recruitment, and neutrophil recruitment) were upregulated in the high OAG score group. In contrast, the activity of killing cancer cells (Step 7) was downregulated (**Figure 7F**). Lastly, it was found that the OAG score was positively correlated with a majority of the expression of immune checkpoint genes, especially *TIM3*, *CD80*, *LAIR1*, and *VTCN1* (**Figure 7G**). Moreover, there existed a close relationship in the expression between *PD-L1*, *PD-1*, *CTLA4*, *LAG3*, *TIM3*, *TIGIT*, *IDO1*, *CD80*, *LAIR1*, and *CD200R1*.

### 3.9 Underlying response of sorafenib, TACE, and chemotherapeutic treatments

**Figure 8:**
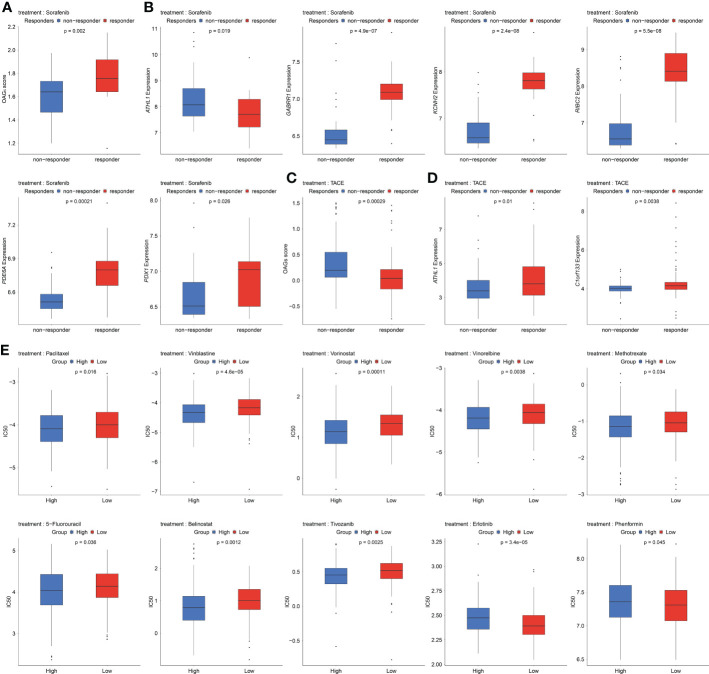
Underlying response of sorafenib, transcatheter arterial chemoembolization (TACE), and potential chemotherapeutic treatment regimens. **(A)** OAG score associated with sorafenib treatment response in GSE109211 dataset. **(B)** A part of OAG (*ATHL1*, *GABRR1*, *KCNH2*, *RIBC2*, *PDE6A*, and *PDX1*) expression also correlated with sorafenib treatment response in GSE109211 dataset. **(C)** Correlation between OAG score and TACE treatment response in GSE104580 dataset. **(D)** ATHL1 and C1orf133 expression correlated with TACE treatment response in GSE104580 dataset. **(E)** The GDSC database analysis revealed that OAG score could distinguish patients potentially sensitive to different chemotherapeutic regimens. OAG, overweight/obesity-associated gene; GDSC, Genomics of Drug Sensitivity in Cancer.

Sorafenib remains the standard of care in the first-line treatments for HCC patients. In the present study, the relationship between the sorafenib responder and the OAG score was then investigated. Noticeably, it was discovered that responders to sorafenib had higher OAG scores compared with those without response (p = 0.002, **Figure 8A**). Regarding the role of each involved OAG, it was noticed that the lower expression of *ATHL1* but higher expression of *GABRR1*, *KCNH2*, *RIBC2*, *PDE6A*, and *PDX1* was significantly correlated with the response of sorafenib treatment in HCC (p < 0.05, **Figure 8B**). Conversely, response assessment for patients treated with TACE treatment showed that responders had markedly lower OAG scores than non-responders (p < 0.001, **Figure 8C**). At the same time, the expression of *ATHL1* and *C1orf133* was positively correlated with the response of TACE treatment in HCC (p < 0.05, **Figure 8D**). In addition, the GDSC database analysis further demonstrated that the predicted IC50 values of paclitaxel, vinblastine, vorinostat, vinorelbine, methotrexate, 5-FU, belinostat, and tivozanib were significantly lower in the high OAG score group (p < 0.05, **Figure 8E**), whereas the predicted IC50 values of erlotinib and phenformin were significantly lower in the low OAG score group (p < 0.05, **Figure 8E**). Overall, the OAG score was of guiding significance in treatment selection.

## 4 Discussions

HCC is a type of malignant cancer with extraordinary heterogeneity, usually accompanied by concomitant multiple molecular heterogeneities in genomic instability, transcriptomic disturbance, and signaling maladjustment. In most cases, HBV/HCV infections or alcohol-induced chronic hepatitis and fibrosis are thought as the major causes contributing to HCC. Nevertheless, the pandemic of overweight/obesity has gradually changed such a circumstance, and a growing body of evidence has demonstrated that overweight and obesity are highly correlated with increased risk and earlier recurrence in HCC (32, 33). Nevertheless, precise molecular mechanisms through which overweight/obesity promotes the development and progression and potentially affects the therapy response of HCC are scarcely known. As a multiplicative interaction between overweight/obesity and alcohol despite low and moderate alcohol intakes, over other risk factors, increases the risk and death due to HCC (34, 35), in the present study, a comprehensive overweight/obesity-associated transcriptome was identified after excluding HCC patients with the alcohol consumption history. Notably, overweight/obesity-associated transcriptome was found to be mainly involved in the metabolic processes, and this overweight/obesity-associated metabolic transcriptome was closely correlated with not only clinical outcome but also the immune microenvironment and immunomodulation in HCC. By the foundation of this, a more robust OAG signature was constructed, whereas clinical association analysis showed that the OAG signature was not correlated with BMI in the whole TCGA-HCC cohort. Regarding non-alcoholic HCC patients, a higher OAG score was associated with a higher proportion of individuals with overweight/obesity (51.01% *vs.* 41.10%), but there was no statistically significant difference either. In most cases, risk factors of viral infection, alcohol, smoking, overweight/obesity, and others did not occur alone in HCC, and usually, they were synergistic risk factors (36, 37). Therefore, multiplicative interaction between risk factors mainly caused clinical features of gender, age, BMI, and others, which were not independent prognostic factors; meantime, it was identified that there was nearly no positive correlation between the OAG signature and BMI. In addition, heterogeneity between different individuals also causes the deviation of BMI; unfortunately, there is a lack of systemic classification methods defining cases of overweight/obesity (38). In the present study, it was identified that the OAG signature was the only independent prognostic factor in three retrospective cohorts, and the OAG signature performed quite well in prognosis prediction for HCC patients, even for early-stage individuals. Moreover, the OAG score was highly correlated with molecular characteristics and the immune microenvironment and had the potential capacity of evaluating the response of sorafenib, TACE, or chemotherapy treatment.

Regarding the novel established the OAG signature, which contained a total of 17 genes and was independent of clinical features in HCC, within the OAG signature, 6 and 11 of these 17 OAGs, respectively, served as protective factors and risk factors at the transcriptomic level. Furthermore, enrichment analysis revealed that identified OAGs were majorly involved in the metabolic processes. In contrast, it should be emphasized that the expression of only *DPYSL4*, *MMP3*, *HTRA3*, *PDX1*, *C21orf29*, and *ATHL1* proteins was ever observed in the cytoplasm/membrane by IHC staining analysis among HCC patients, of which the expression of *DPYSL4*, *MMP3*, and *ATHL1* proteins was clearly detected in all involved samples. As reported, *DPYSL4* was associated with glycolysis (39) and hypoxia (40) in HCC, and meanwhile, its overexpression was proved to be correlated with the progression and metastasis of HCC. *MMP3*, encoding a kind of protein as a member of matrix metalloproteinase, was well known to be involved in tumor progression and invasion (41), while specific peptide inhibitors targeting MMP3 could suppress HCC cell migration (42). In contrast, the function or role of *HTRA3*, *PDX1*, *C21orf29*, or *ATHL1* in HCC was still unknown, and it was the first time that this is revealed in the present study that their expression was significantly correlated with prognosis. Of note, it should be highlighted that *ATHL1* expression was correlated with prognosis and performed well in prognosis prediction. More impressively, downregulation and upregulation were significantly associated with sorafenib and TACE treatment response, respectively. *ATHL1*, encoding a protein-glucosyl-galactosyl-hydroxylysine glucosidase (PGGHG), was mainly involved in the carbohydrate metabolic process, and three carboxyl residues, Asp301, Glu430, and Glu574, were responsible for the functional role of PGGHG (43). Altogether, it could be inferred that inhibition of *ATHL1* expression or PGGHG activity before sorafenib treatment might improve the therapeutic response. In addition, the IHC staining of the expression of *GABRR1*, *GAGE2D*, *KCNH2*, *PRTG*, *SHC4*, and *SMIM32* proteins was unknown and not reported in the HPA database and, hence, needs further investigations at the protein level. In our study, the expression of *GABRR1*, *GAGE2D*, *KCNH2*, *PRTG*, *SHC4*, and *SMIM32* was significantly correlated with OS of HCC, and the expression of *GABRR1* and *KCNH2* was associated with sorafenib treatment response. *C1orf133* was known as a kind of ncRNA *SERTAD4-AS1*, and also its expression was first identified in our study to be correlated with prognosis of HCC and even associated with TACE treatment response.

Dysregulation of hepatic metabolisms, such as oxidative phosphorylation, glycolysis, and fatty acid metabolism, was critical to the development and progression of liver disease, especially in patients with non-alcoholic hepatitis disease (44, 45). Similarly, GO and KEGG pathway enrichment disclosed that the aberrantly regulated biological activities associated with the OAG score in the present study were abundantly enriched with genes involved in the cytochrome P450-mediated metabolism, fatty acid metabolism, amino acid metabolism, retinol metabolism, and xenobiotic metabolism. Cytochrome P450-mediated metabolism usually caused the accumulative reactive oxygen species (ROS), including superoxide anion, hydrogen peroxide, and hydroxyl radical, which played a key role in contributing to steatohepatitis (46) and promoting invasiveness of HCC cells (47). The dysregulation of fatty acid metabolism might directly result in the anomalous activities of peroxisome proliferation-activated receptors (PPARs: α, β, γ) and related signaling pathways, which acted as fatty acid sensors (48). Moreover, these PPAR members were critical transcription factors regulating mitochondrial functions and energy homeostasis (49); thus, some pharmacological strategies of PPAR agonists have emerged and are associated with improved clinical outcomes (50). Moreover, perturbation of amino acid metabolism was also correlated with the progression of hepatic live diseases (51). Noticeably, cepharanthine treatment could inhibit HCC cell proliferation and migration by regulating amino acid metabolism (52). Of note, hepatic tissue in individuals stores almost 70% of retinoids (53); as reported, the inhibition of retinoids or the loss of hepatic retinoid signaling potentially leads to oxidative stress (54), which was associated with the progression of liver diseases (55). Moreover, retinoids were involved in many biological activities, including apoptosis promotion and inflammation response; altogether, retinol metabolism was markedly correlated with the development and progression of HCC (56). In addition, Hallmark pathways of oxidative phosphorylation and cell cycle/DNA replication-related signaling were abundantly enriched in the HCC patient group with inferior survival further confirming that increased oxidative stress/oxidative phosphorylation significantly promoted the progression of HCC (57). Furthermore, oxidative phosphorylation activation was also correlated with chemotherapeutic resistance (58). Overall, dysregulated metabolisms associated with the OAG score enormously affected clinical outcomes and immunomodulation or inflammatory regulation in HCC.

From another aspect, genomic characterization can offer a compelling framework to demonstrate the functional significance and discover key genes stimulating the development and progression of HCC. Nevertheless, evidence is mounting that more and more therapeutic regimens targeting on-oncogene alterations are engendered, compared to the tumor suppressor genes or recurrently altered passenger genes (59). However, there were only a few disparities in the genomic characterizations between the high and low OAG score groups. Despite that *TP53* and *DNAH10* frequently altered in the high OAG score groups, no specific alteration sites of *TP53* or *DNAH10* were significantly more prevalent. According to the Catalogue of Somatic Mutations in Cancer database, over 30% of all HCC patients harbored at least one alteration in *TP53*, ranking first in terms of alteration frequency in HCC. As a tumor suppressor gene, *TP53* alterations were expectedly correlated with the development of progression of HCC (60), and consistent with this, HCC patients with high OAG scores had more altered *TP53* and inferior OS. Generally, cells with altered TP53 protein could escape from apoptosis and gradually develop into HCC cells due to DNA damage events, which could also contribute to HCC progression (61). Moreover, *DNAH10* alteration was positively correlated with the OAG score, and it was found that patients harboring *DNAH10* alterations had significantly worse OS in HCC, compared with wild-type patients. *DNAH10*, namely, dynein axonemal heavy chain 10, encodes a protein of inner arm dynein heavy chain (62); however, the role of *DNAH10* in liver tissue is scarcely known. In contrast, there were several studies revealing that altered *DNAH10* was correlated with the elevated level of high-density lipoprotein cholesterol (63), adipocyte function (64), and adipocyte differentiation (65). Based on the experiment of RNAi-knockdowns for *DNAH1* expression in *Drosophila*, the total triglyceride levels were elevated within the body (66). Altogether, it could be implied that altered *DNAH10* might aggravate the progression of HCC by influencing lipid metabolism, which needed further experimental validation. Interestingly, there existed two cases of *CTNNB1-AXIN1* and *CTNNB1-TP53* exhibiting mutually exclusive alterations in the low OAG score group, suggesting that their effects in the same pathway were probably redundant and that there was an epistatic association between these two genes; however, this phenomenon did not occur in the high OAG score group.

Multi-kinase inhibitors, such as sorafenib and lenvatinib, are still the first-line treatment, while immune checkpoint blockades, alone or in combination with other regimens, have revolutionized the clinical management and treatment of HCC (67). Nevertheless, the molecular mechanisms influencing immune response and evasion in HCC remain to be fully elucidated. Impressively, it was initially identified that overweight/obesity-associated transcriptome in the present study was markedly associated with immunomodulation and the immune microenvironment of HCC. Moreover, most chemokines, receptors, immunomodulators, and MHC molecules were upregulated in the high OAG score group, implying that a high OAG score potentially had higher activity in antigen presentation and processing as well as the promoting recruitment of antigen-presenting cells, CD8+ T cells, and Th17 cells. Comparatively, the cancer immunity cycle was a more comprehensive reflection of the immunomodulation system, representing the immune response to tumors (21). Controversially, the activity of killing cancer cells (Step 7) was downregulated in the high OAG score group, which presented with a higher level of inflamed TME and increasing activity in both the releasing of cancer cell antigens (Step 1) and part of the trafficking of immune infiltrating cells to tumor cells (Step 4). This discordance might be due to the positive association between the OAG score and PD-L1/PD-1 expression as well as a majority of immune checkpoint gene expression, indicating that these immune checkpoints would suppress cancer immunity and lead to immune evasion (68). In addition, the high OAG score group had a higher level of TIDE score, which has been proven to be negatively correlated with the infiltration of effective CD8+ T cells within tumors (19). Altogether, it was reasonably believed that the final activity of anti-cancer immunity might be downregulated in the high OAG score group. In summary, we strongly recommended that immunosuppressive factors should be inhibited first to prevent the exclusion of T cells from infiltrating tumors (69), which could improve the response of immunotherapy in the high OAG score group. However, immunotherapy was probably not suitable for HCC patients with a low OAG score because of a low level of inflamed TME and immune checkpoint gene expression.

Reversely, over-inflammation in the high OAG score group could substantially stimulate the progression of HCC, while targeting inflammation could become a promising treatment strategy for these patients (70). Sorafenib, having been approved by Food and Drug Administration as the standard treatment for HCC (71), could inhibit inflammatory pathways and reduce liver fibrosis in cirrhotic rats (72). Consistently in the present study, a higher OAG score was significantly correlated with the response to sorafenib treatment, probably owing to the higher level of inflamed TME among these patients. As exhibited, Macrophage M0 was abundantly enriched in the high OAG score group. Compared to the single drug sorafenib for HCC patients, depletion of macrophages by zoledronic acid or clodrolip in combination with sorafenib resulted in the stronger inhibition of HCC progression, angiogenesis, and even lung metastasis (73). Therefore, a combination treatment of sorafenib and zoledronic acid or clodrolip seemed to be more effective for patients with a high OAG score. In addition, it was further identified that the OAG score was negatively correlated with the response to TACE treatment. Regarding the relatively early-stage HCC patients, as suggested, patients with a lower OAG score associated with a lower level of inflamed TME are likely to receive the TACE treatment. Overall, it was demonstrated that the OAG score also had the potential to become a reliable and robust predictor for the response of sorafenib or TACE treatment, which would greatly help promote clinical management and precision medicine for HCC patients. The GDSC data analysis revealed that patients in the high OAG score group were likely to have a higher sensitivity to chemotherapy *via* the drugs paclitaxel, vinblastine, vorinostat, vinorelbine, methotrexate, 5-FU, belinostat, and tivozanib, whereas those in the low OAG score group seemed to be more sensitive to erlotinib and phenformin. However, it needed to be proposed that the evaluation of chemotherapeutic sensitivity was mainly based on pharmacogenomic analysis in cancer cells (74), so further investigations in animal models or clinical trials are needed for verification.

The comprehensive overweight/obesity-associated metabolic transcriptome was profoundly correlated with clinical outcome, immunomodulation, and the immune microenvironment, and afterward, the novel constructed OAG signature could function as an effective independent predictor of prognosis and determine the molecular characterization and TME of HCC, as well as predict the response of sorafenib and TACE treatment. In contrast, there still existed some limitations that should be noted. First, this study was mainly based on the public database and more likely as a retrospective cohort analysis; thus, prospective studies are needed for validation. Second, it was powerful to use machine learning for the construction of the OAG signature, but the bioinformatics analysis still predominated in this process, and it might hinder the clinical significance of some overweight/obesity-associated genes in HCC. Because of this, we found that the OAG score was not significantly associated with BMI; thus, the OAG signature might lack the power to distinguish HCC patients from overweight/obesity patients. However, an in-depth investigation of overweight/obesity-associated transcriptome provided more information about molecular characteristics, the immune microenvironment, and therapy response. Finally, we indirectly evaluated the underlying response of immunotherapy, and HCC patients treated with immunotherapy were not really verified in our study, so more clinical trials should be designated for further exploration. Overall, it might be concluded that transcriptomic characterization driven by overweight/obesity (or higher BMI) played a vital role in the progression of HCC meanwhile, which was also highly associated with the immune microenvironment and therapy response.

## 5 Conclusions

The findings in the present study first disclosed that comprehensive overweight/obesity-associated metabolic transcriptome was significantly correlated with prognosis and TME of HCC, and a novel constructed OAG signature exhibited better performance in prognosis prediction. Moreover, the OAG signature was also associated with the response of sorafenib, TACE, or chemotherapy. This study could offer a clinically applied tool to promote the management of HCC and increase the need for a clear strategy of precision medicine in HCC.

## Data availability statement

The original contributions presented in the study are included in the article/**Supplementary Material**. Further inquiries can be directed to the corresponding author.

## Author contributions

N-NF and B-MY proposed and designated this work. N-NF and X-YD collected and processed the raw data. N-NF, X-YD and Y-SZ conducted the bioinformatics analysis. Y-SZ, Z-KJ and X-HW prepared and made visualizations and were responsible for figures and tables. N-NF and X-YD wrote the original manuscript. N-NF and B-MY revised the manuscript. All authors contributed to the article and approved the submitted version.
