# Supplementary figures and images for: Overweight/obesity-related transcriptomic signature as a correlate of clinical outcome, immune microenvironment, and treatment response in hepatocellular carcinoma
Source: Front Endocrinol (Lausanne). 2023 Jan 12;13:1061091. doi: 10.3389/fendo.2022.1061091 (PMC9877416; doi:10.3389/fendo.2022.1061091)

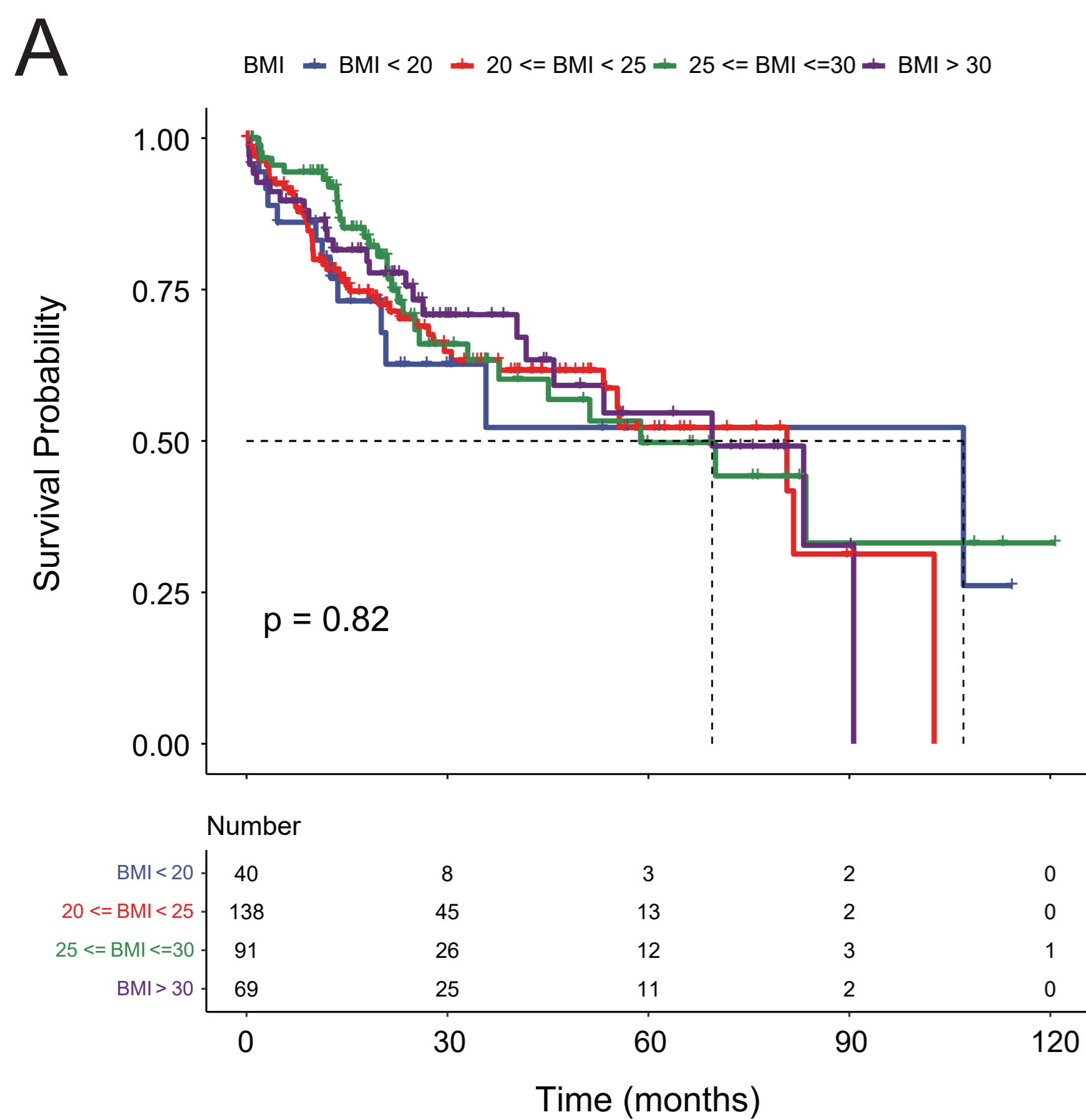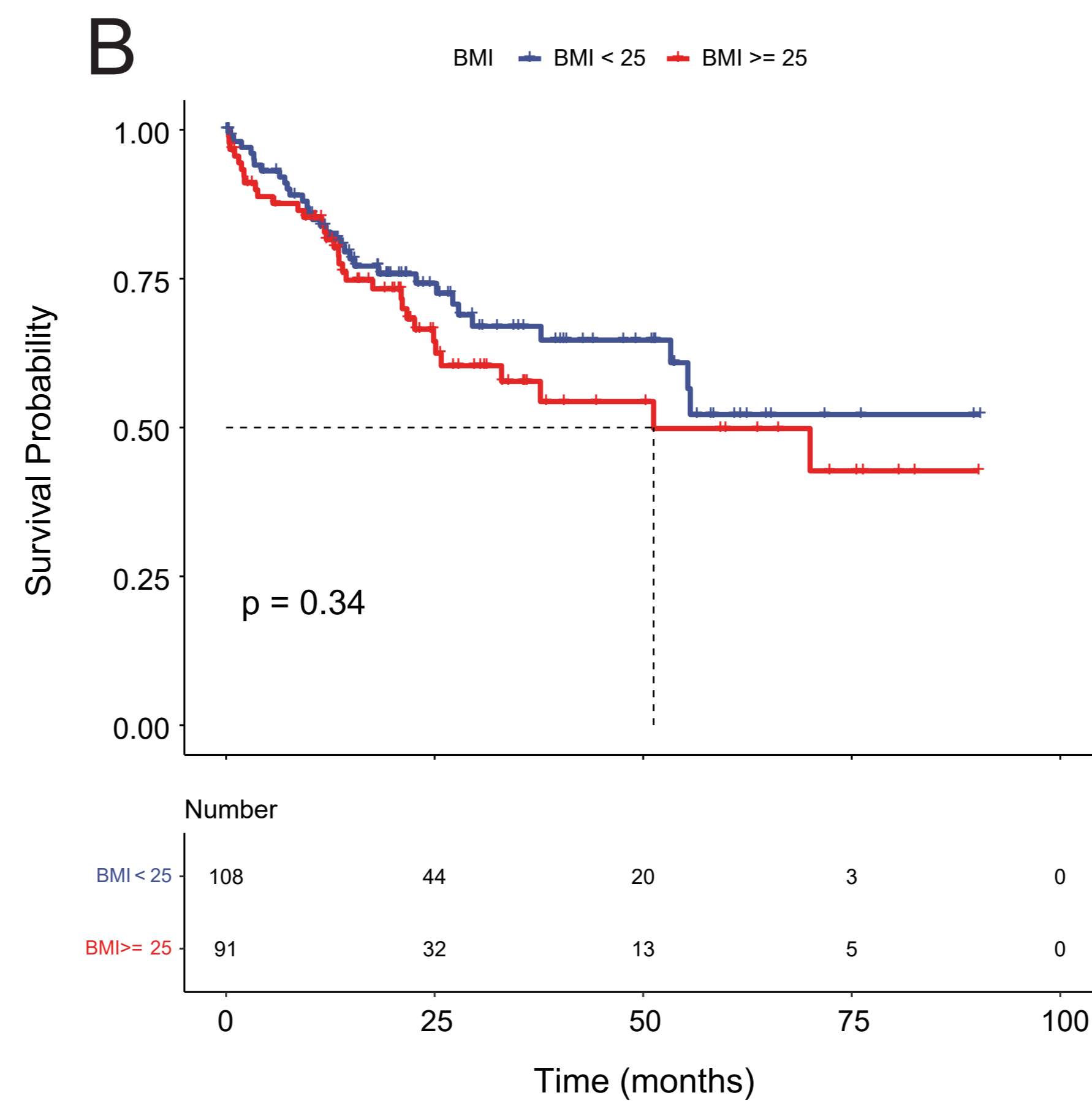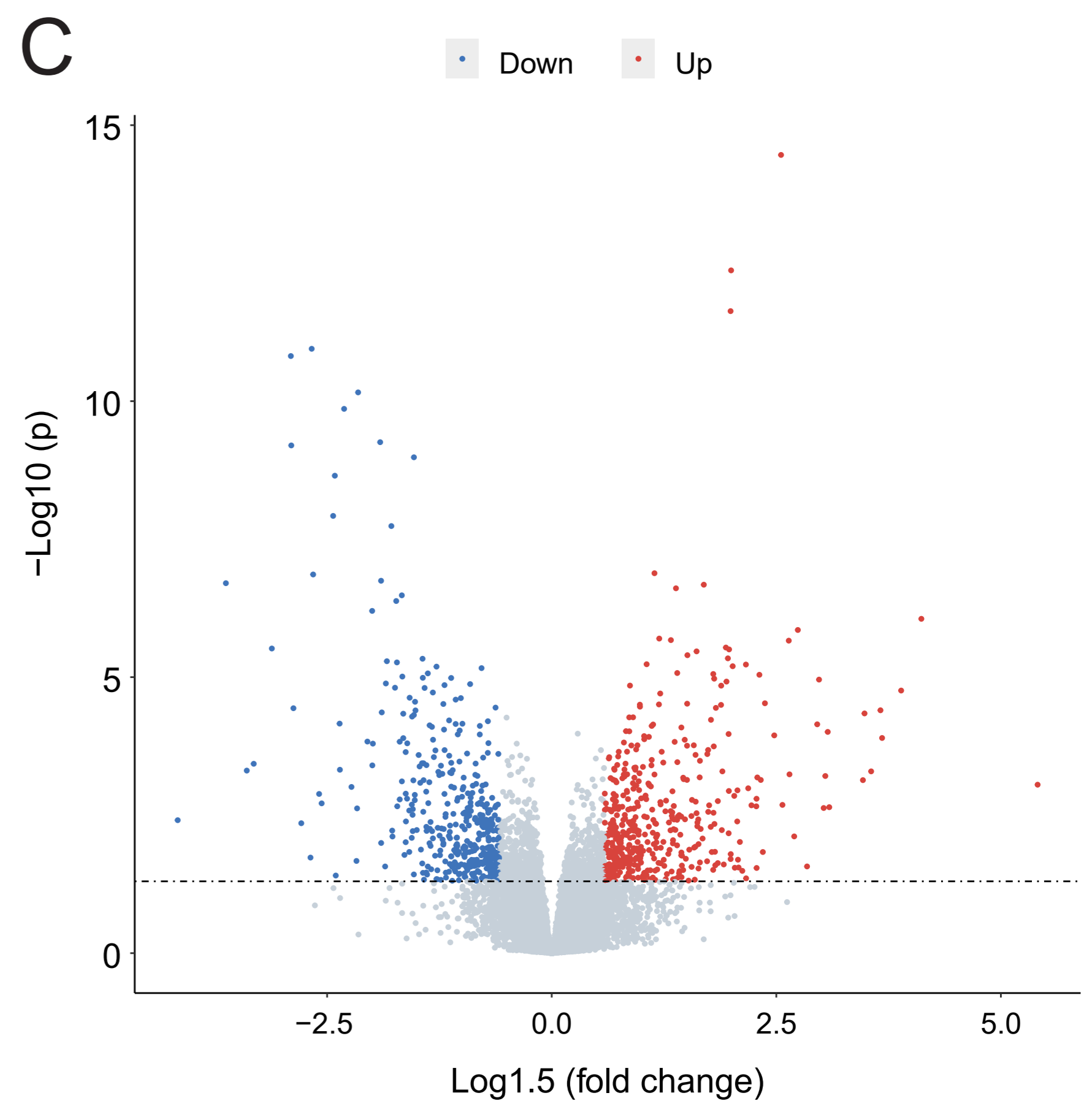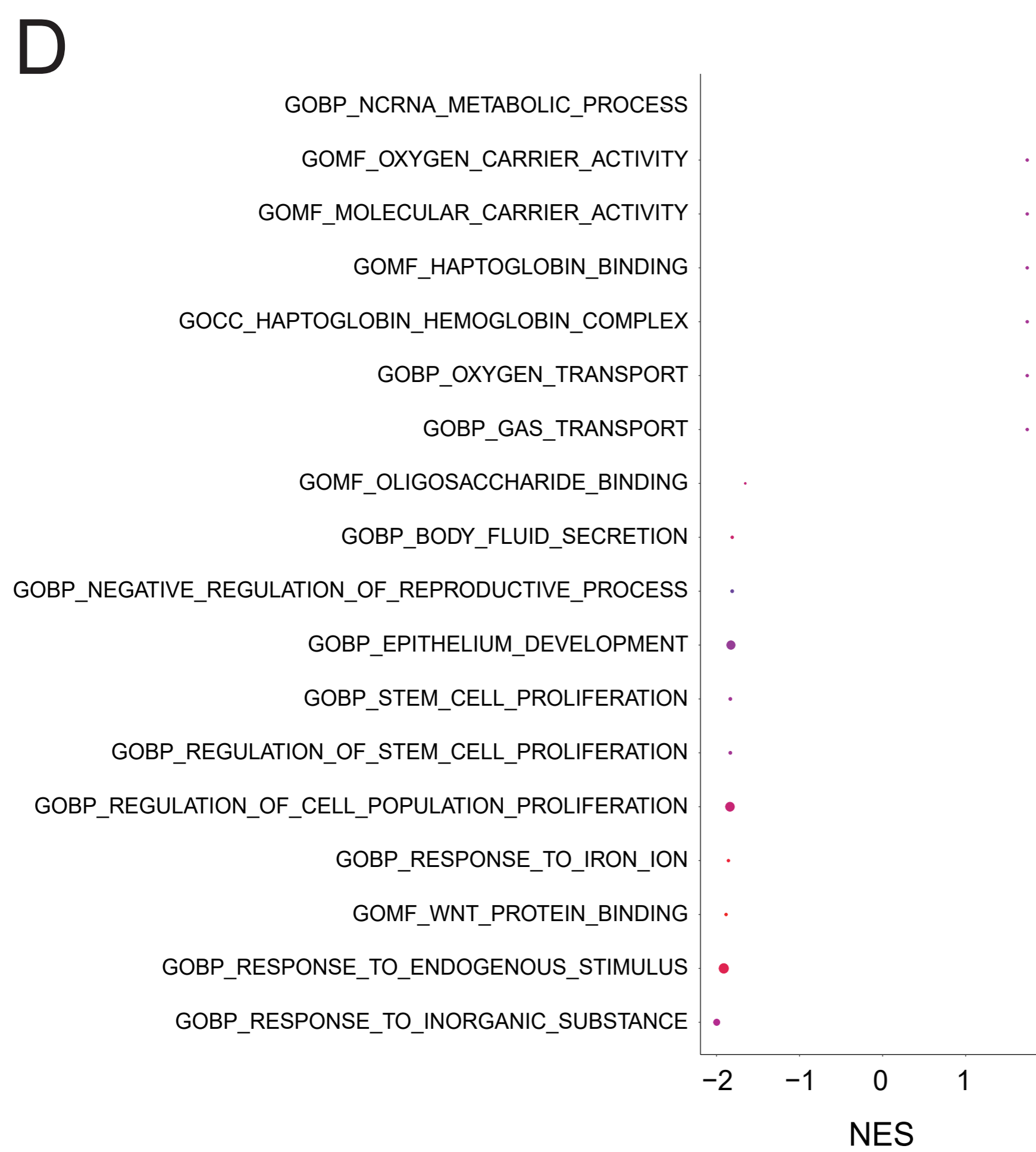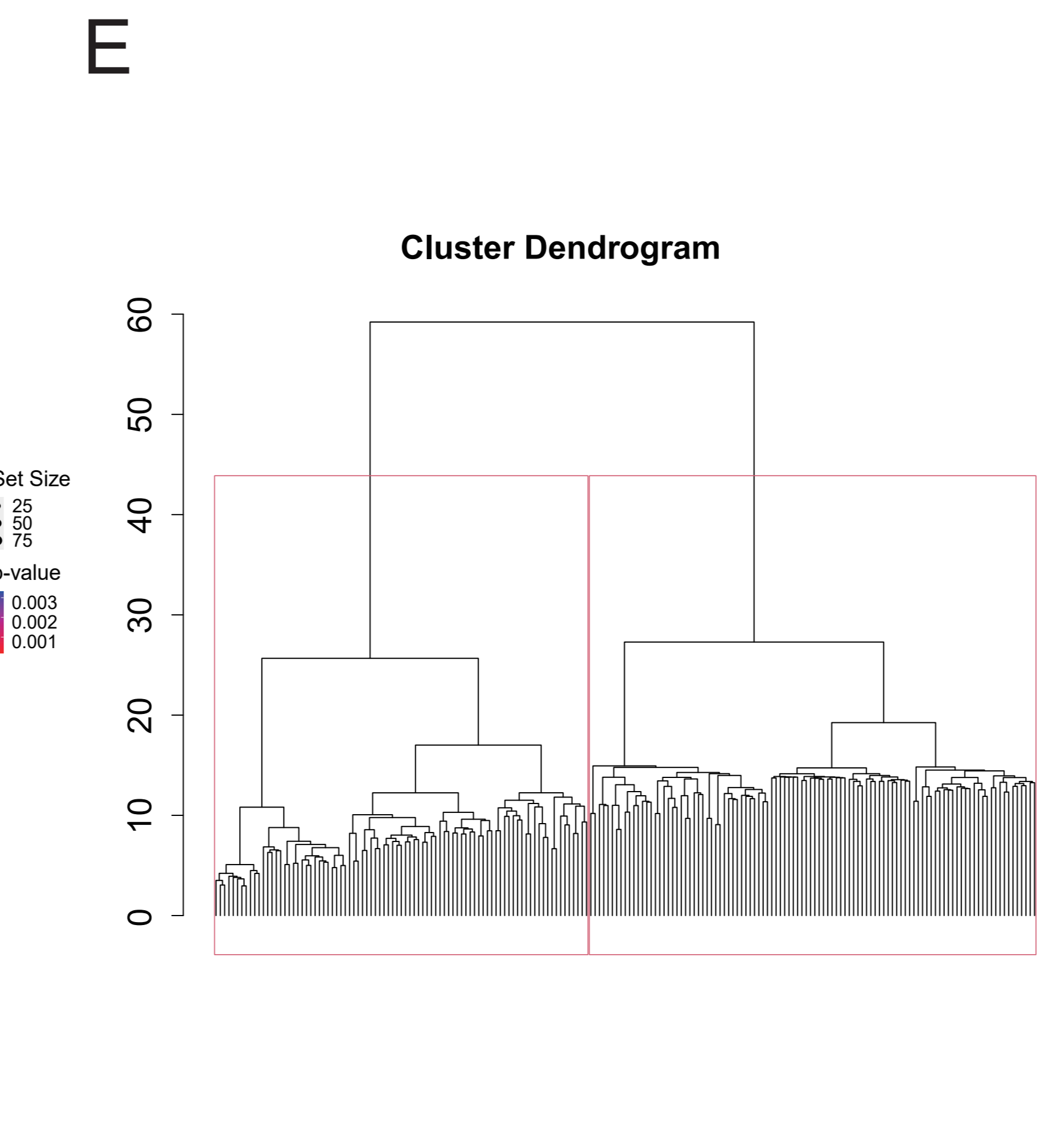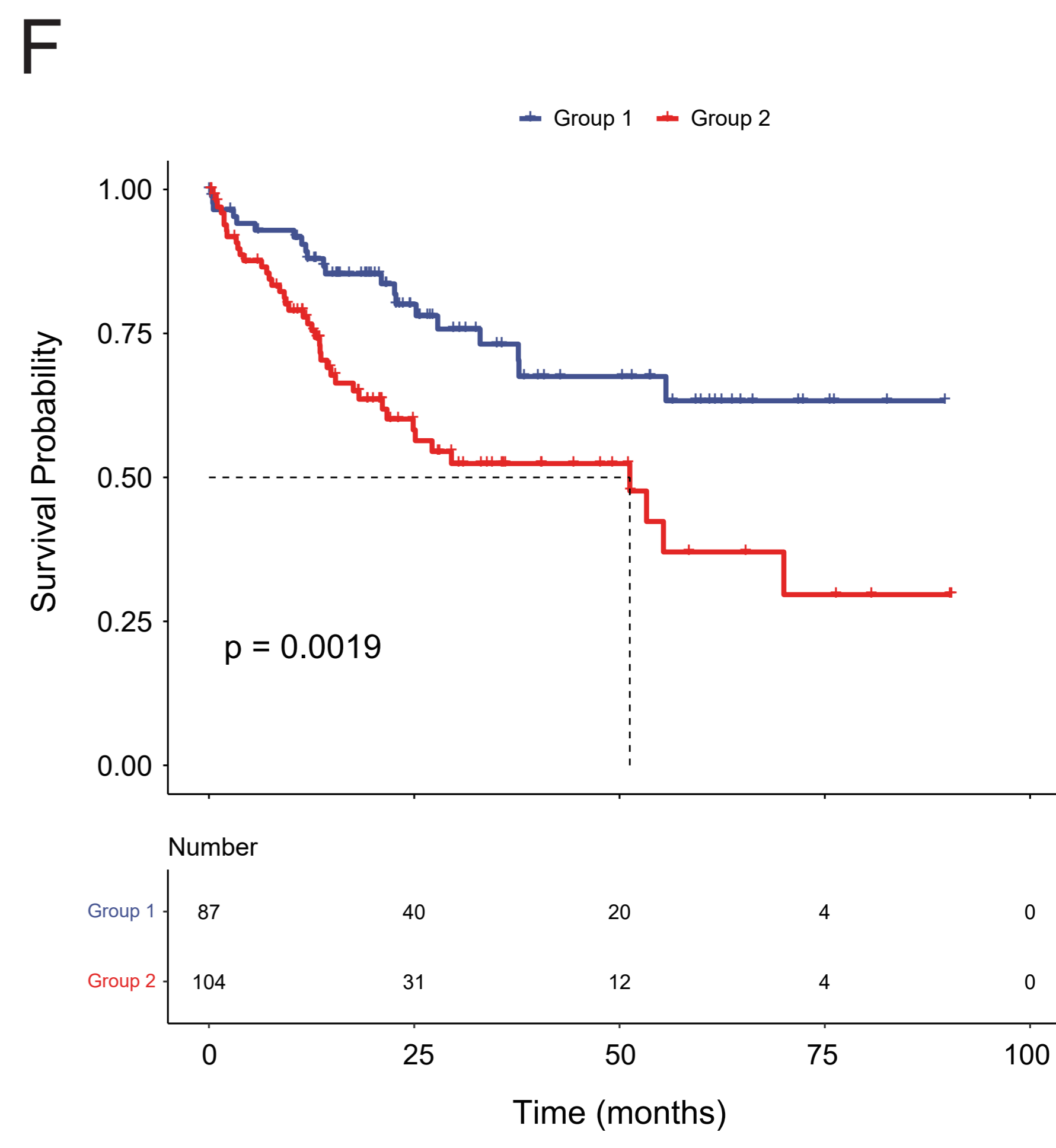

Supplement: Supplementary file 1 [file Image_1.pdf]

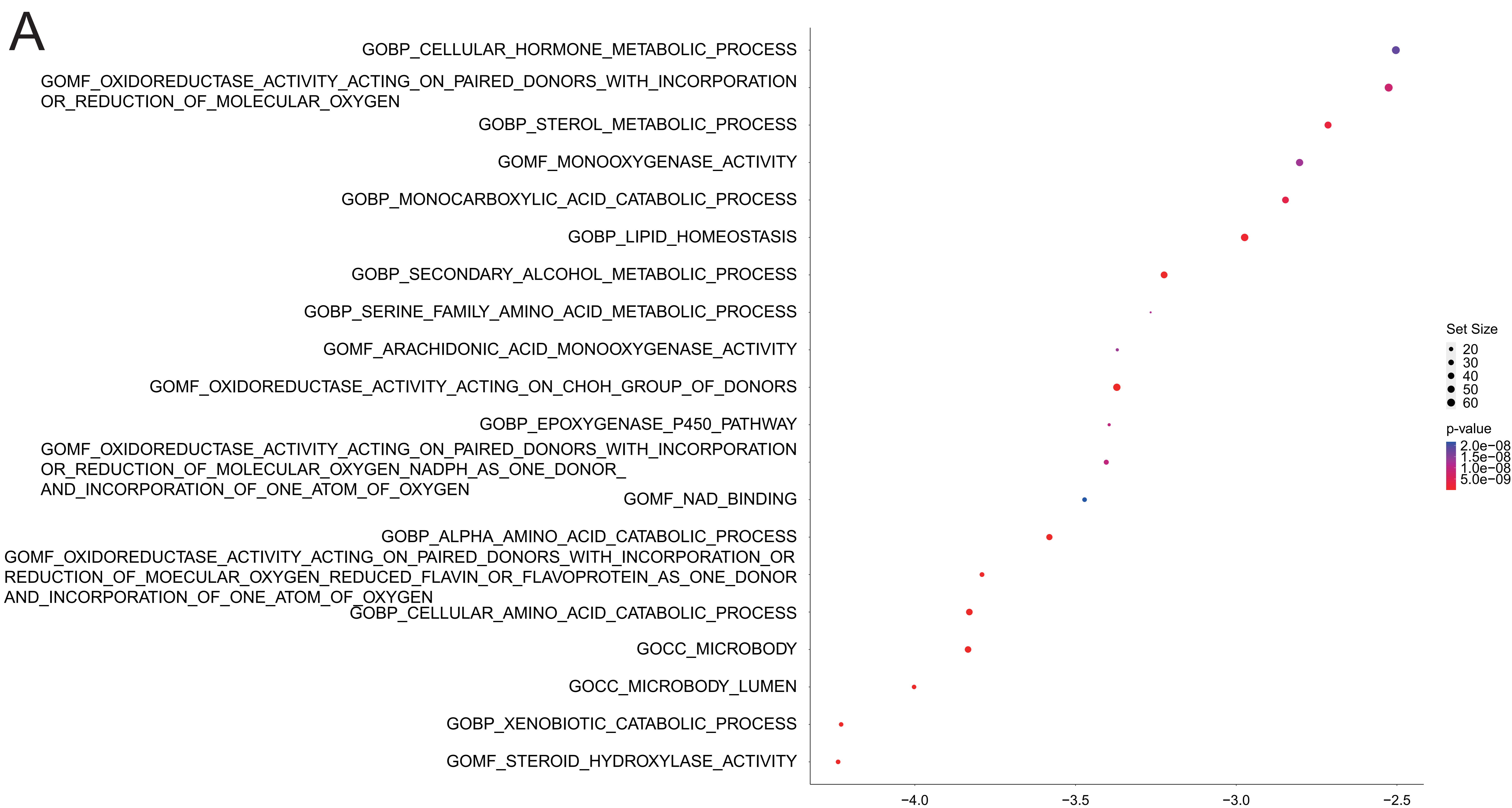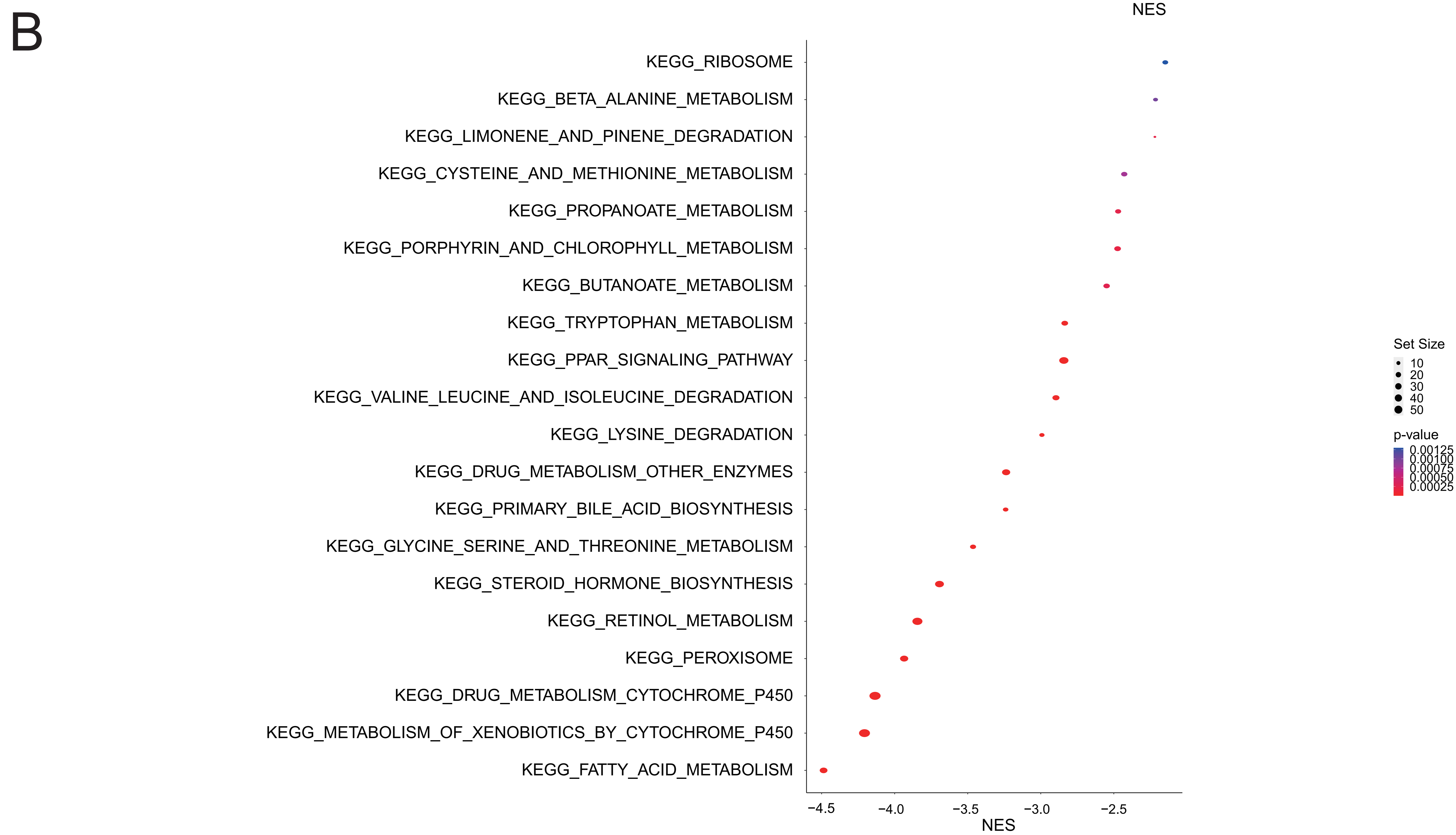

Supplement: Supplementary file 2 [file Image_2.pdf]

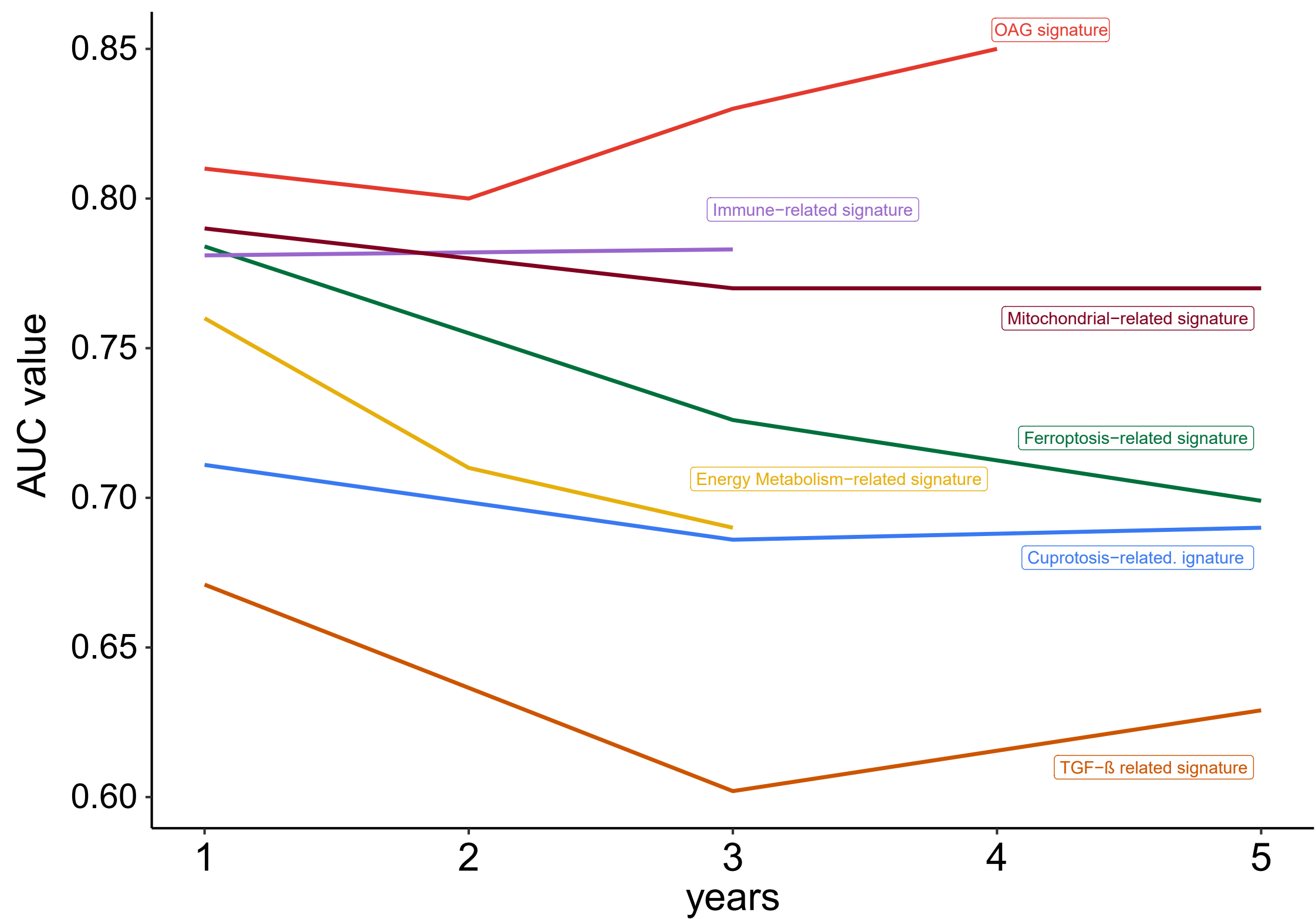

Supplement: Supplementary file 3 [file Image_3.pdf]

HCC patients without alcohol consumption

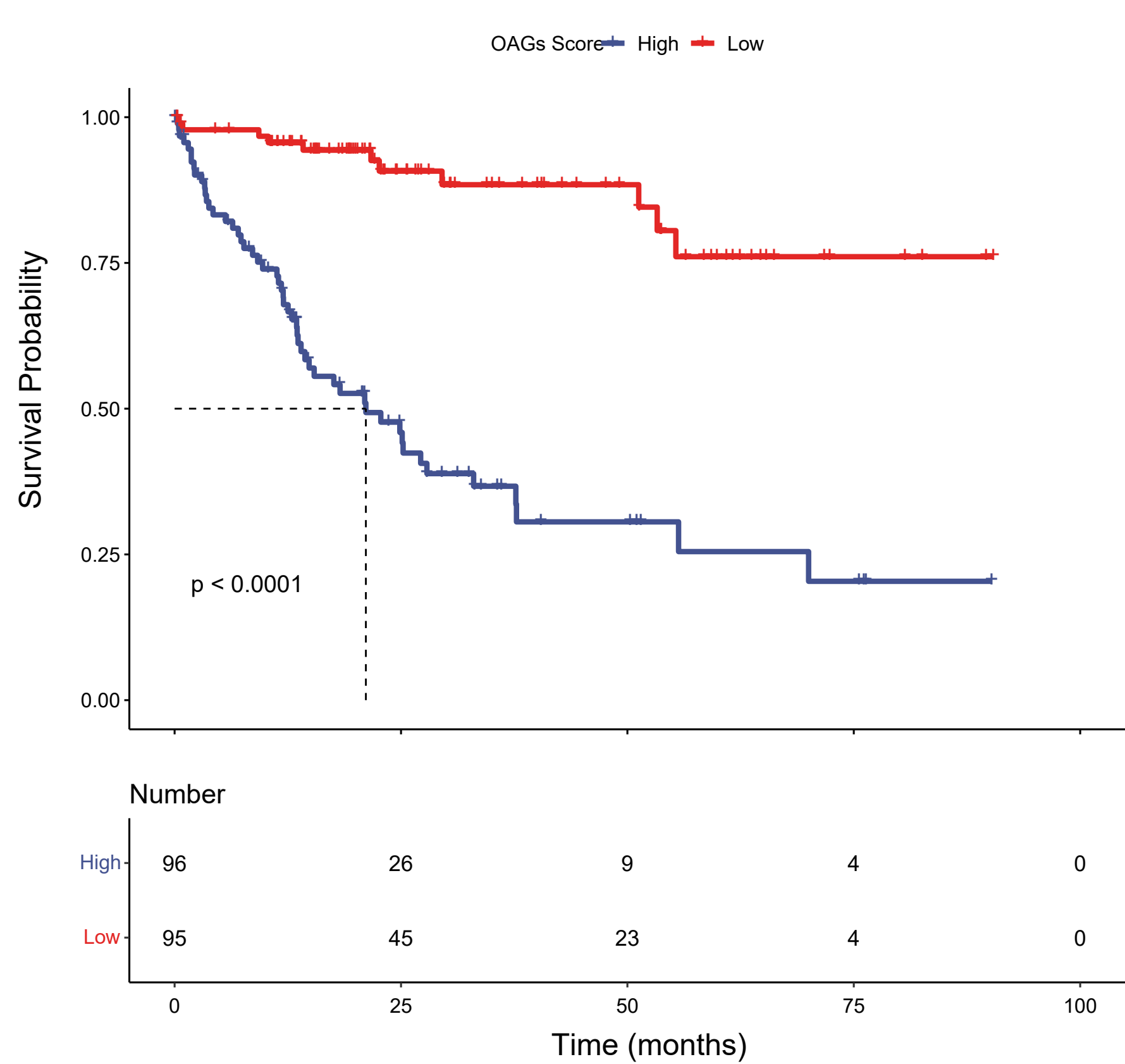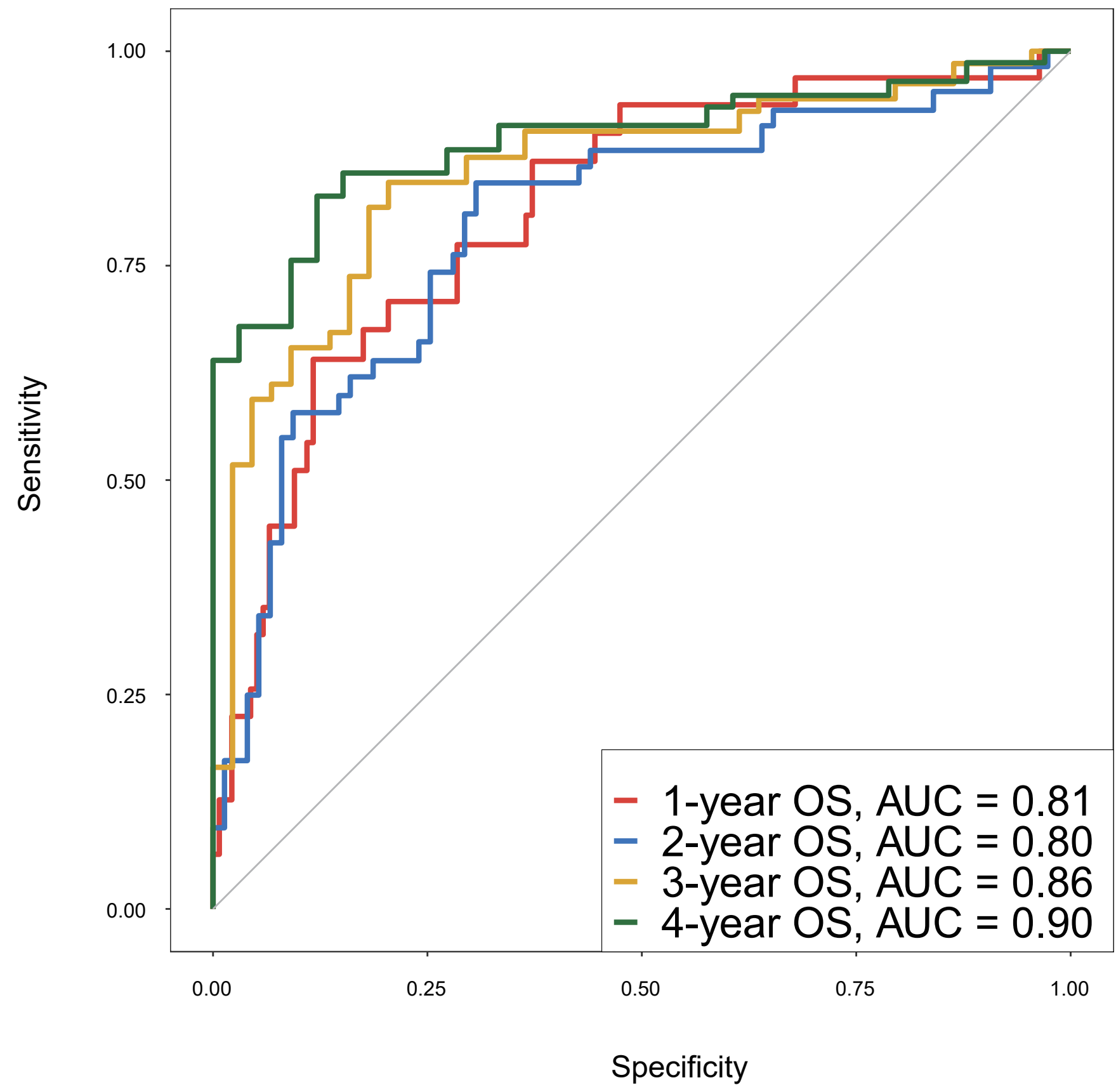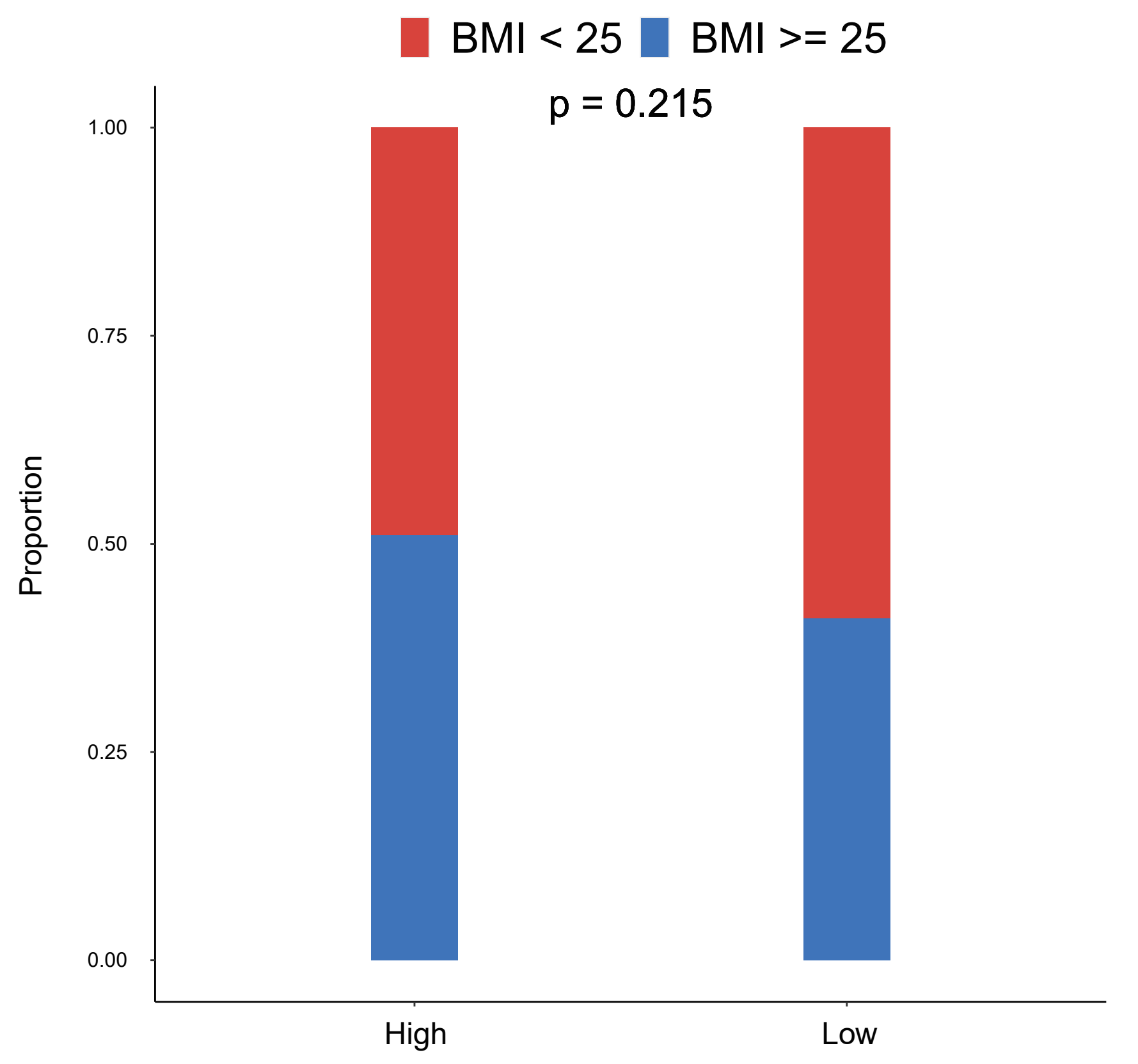

Supplement: Supplementary file 4 [file Image_4.pdf]

1-year ROC curve

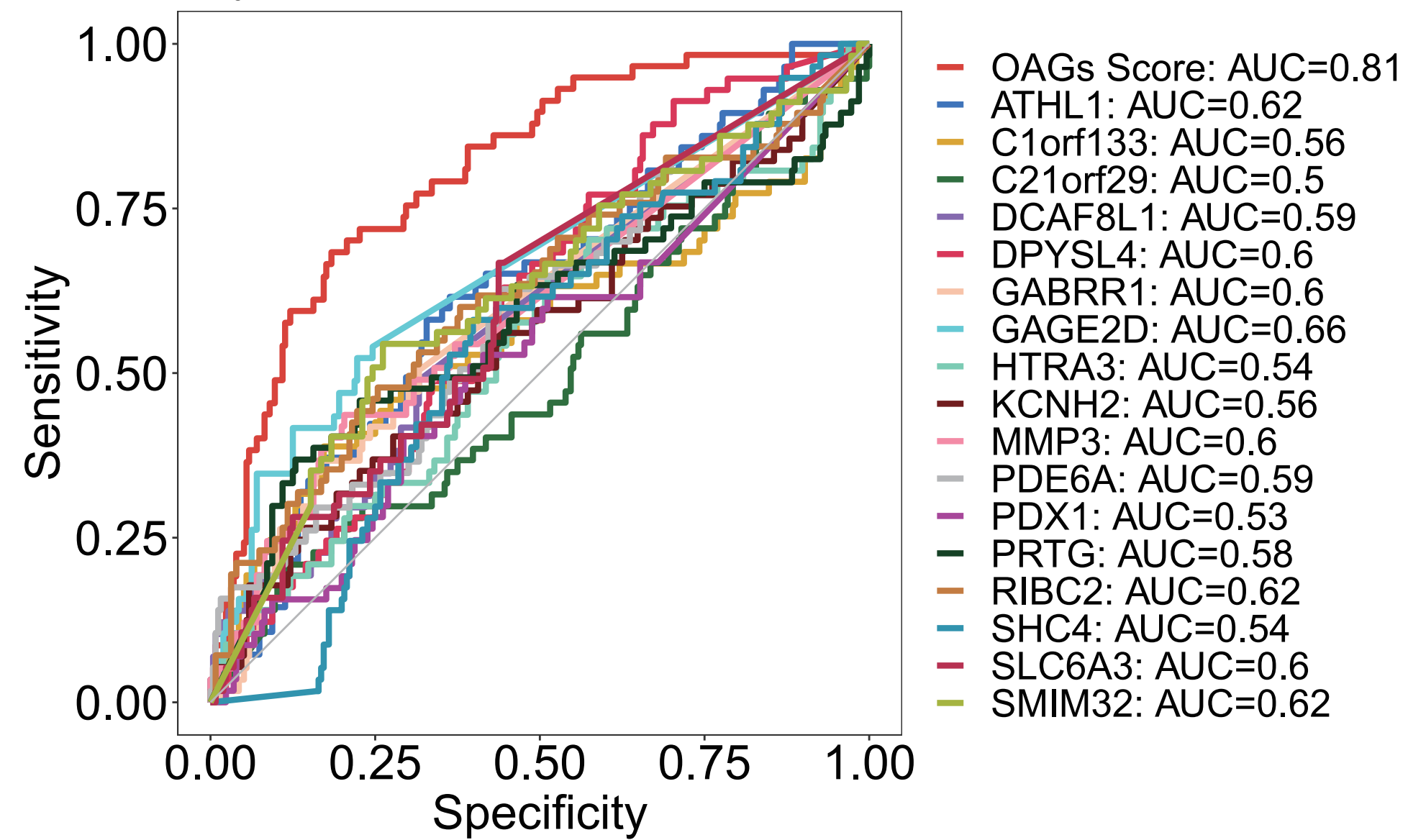

2-year ROC curve

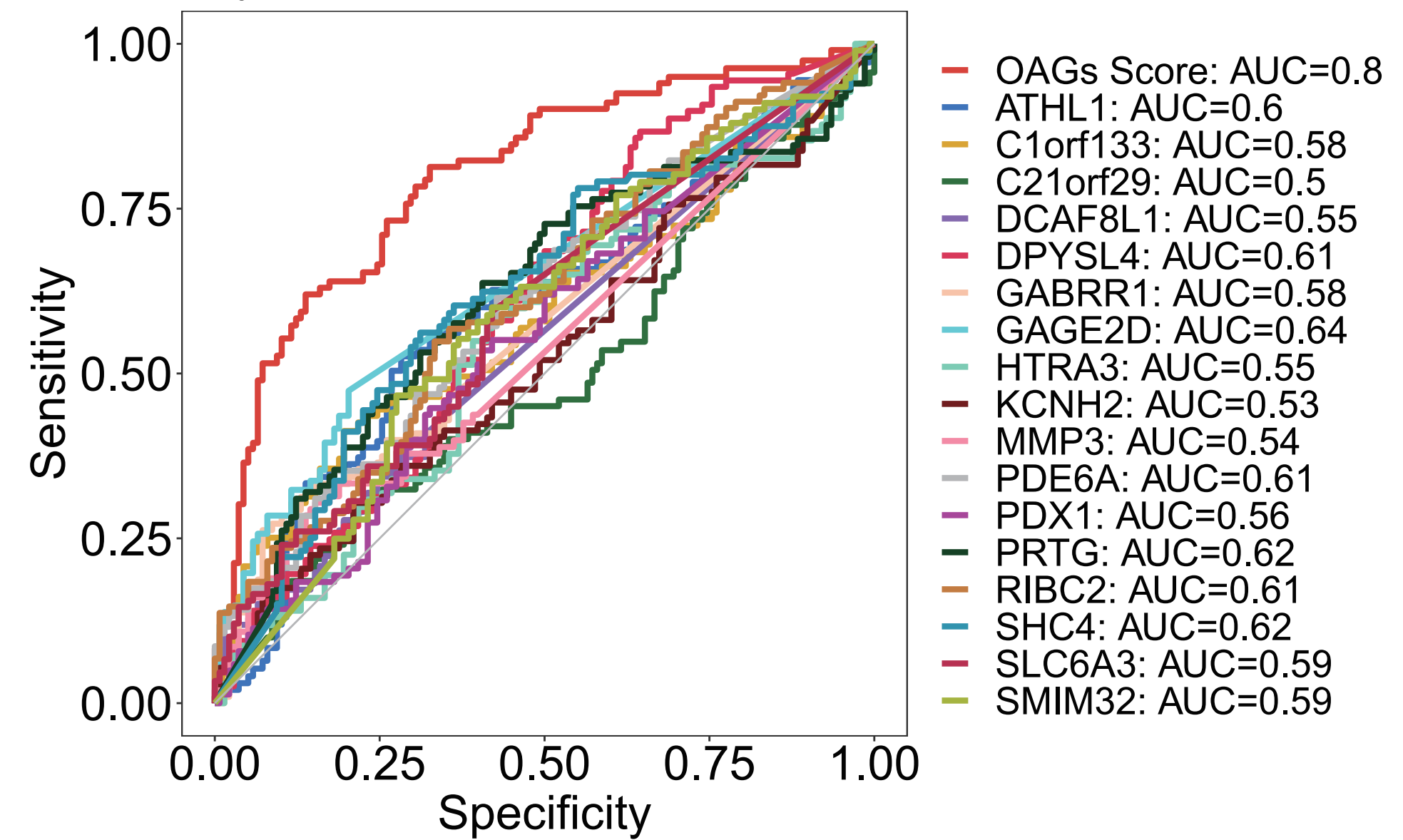

3-year ROC curve

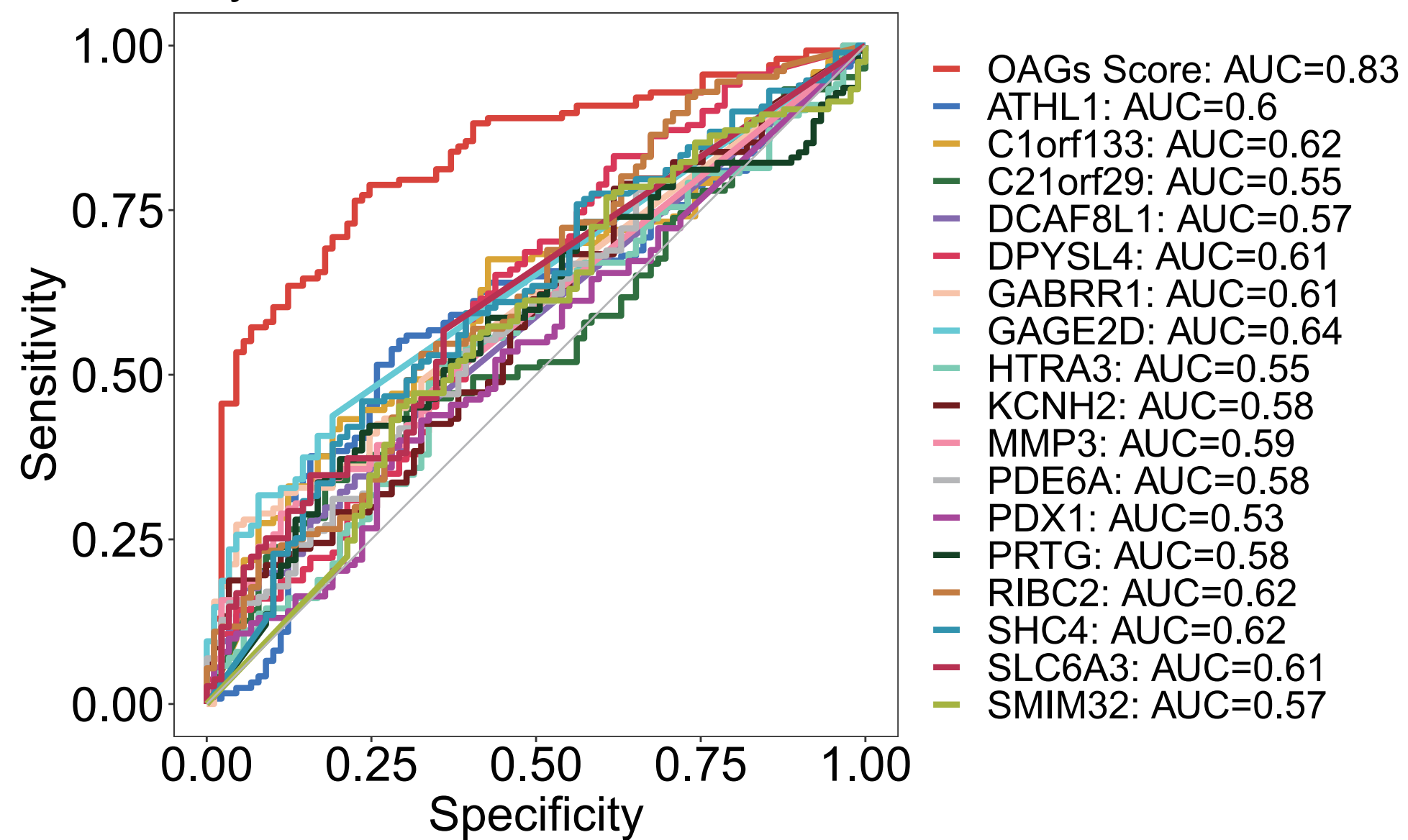

4-year ROC curve

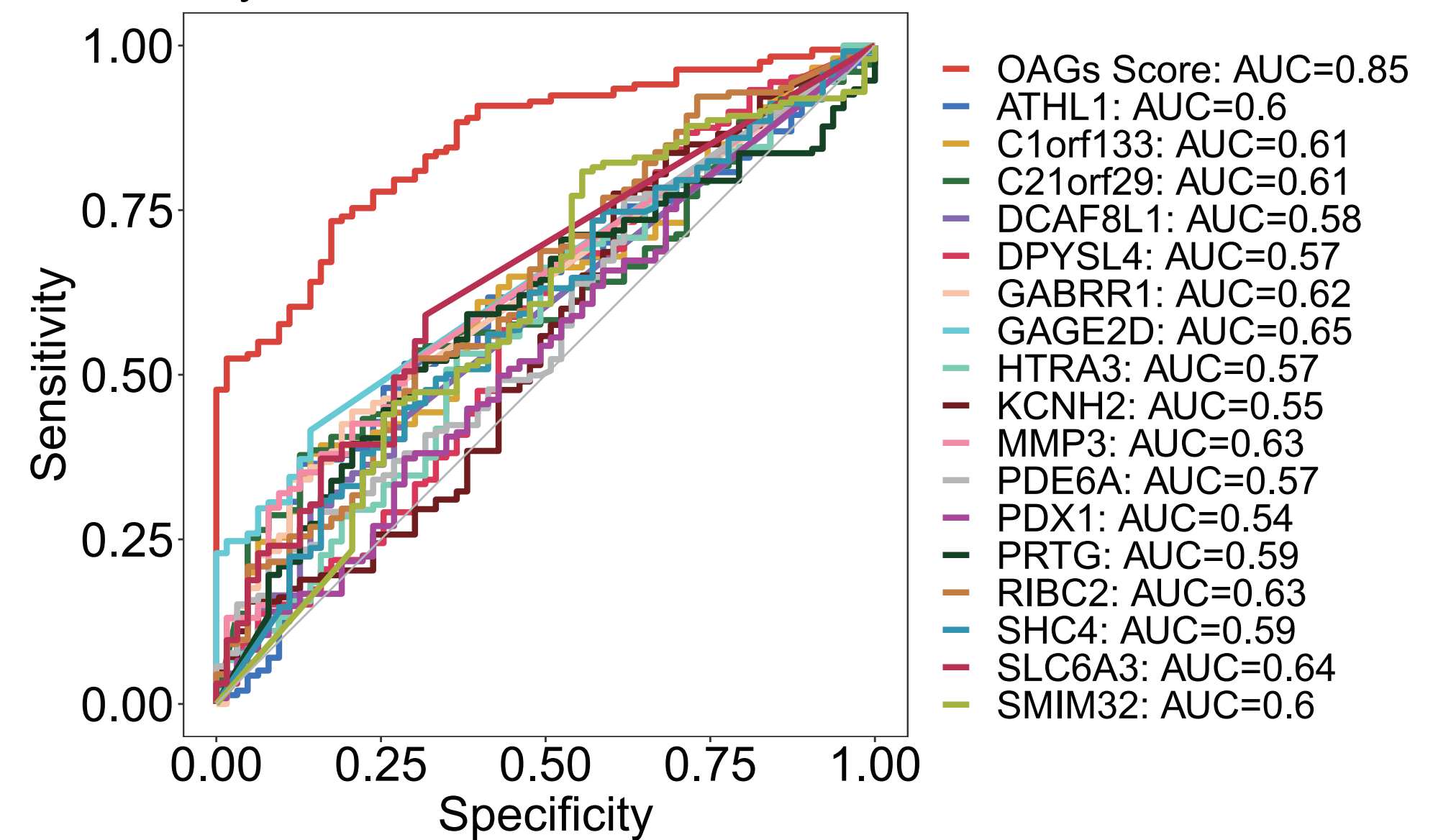

Supplement: Supplementary file 5 [file Image_5.pdf]

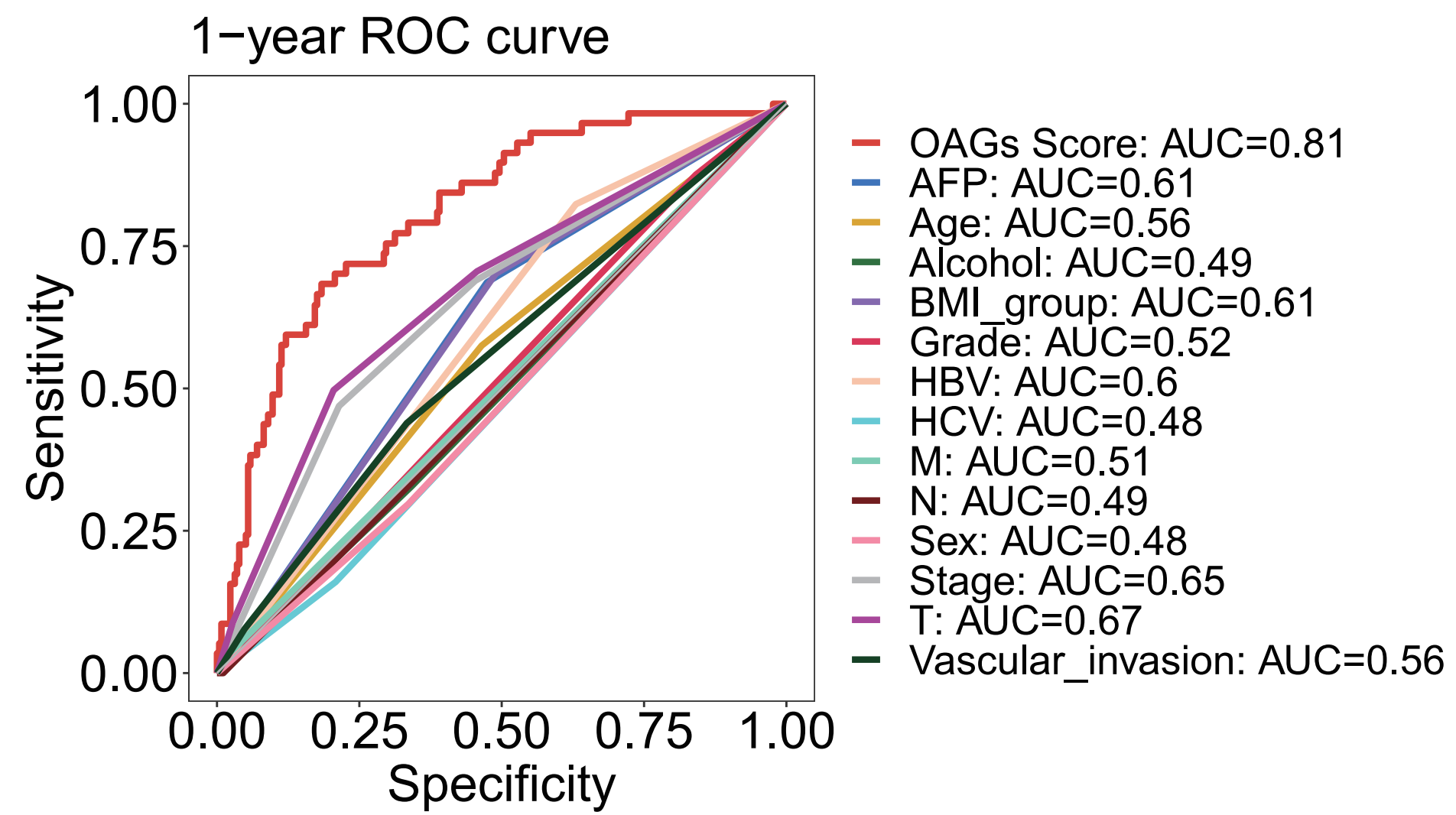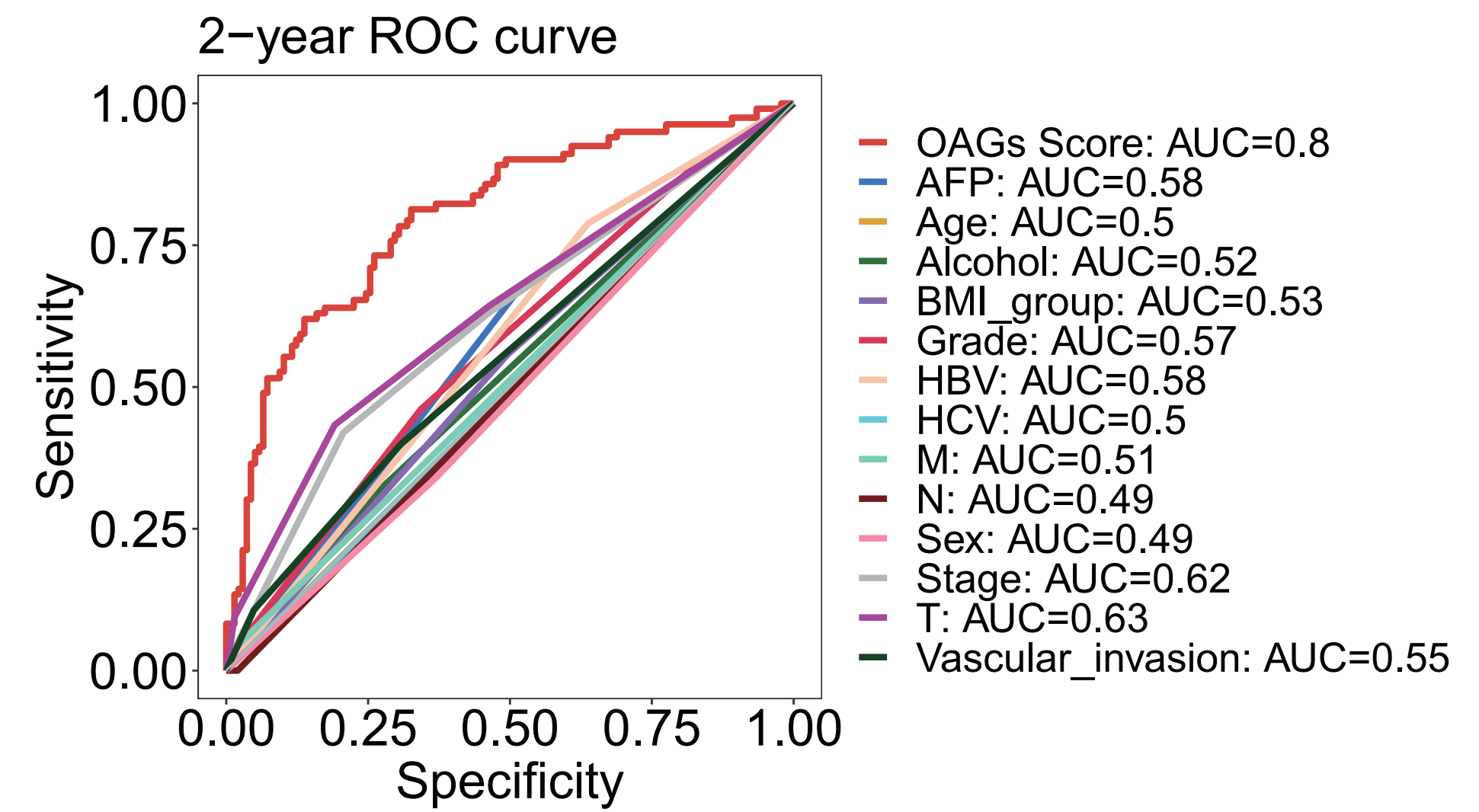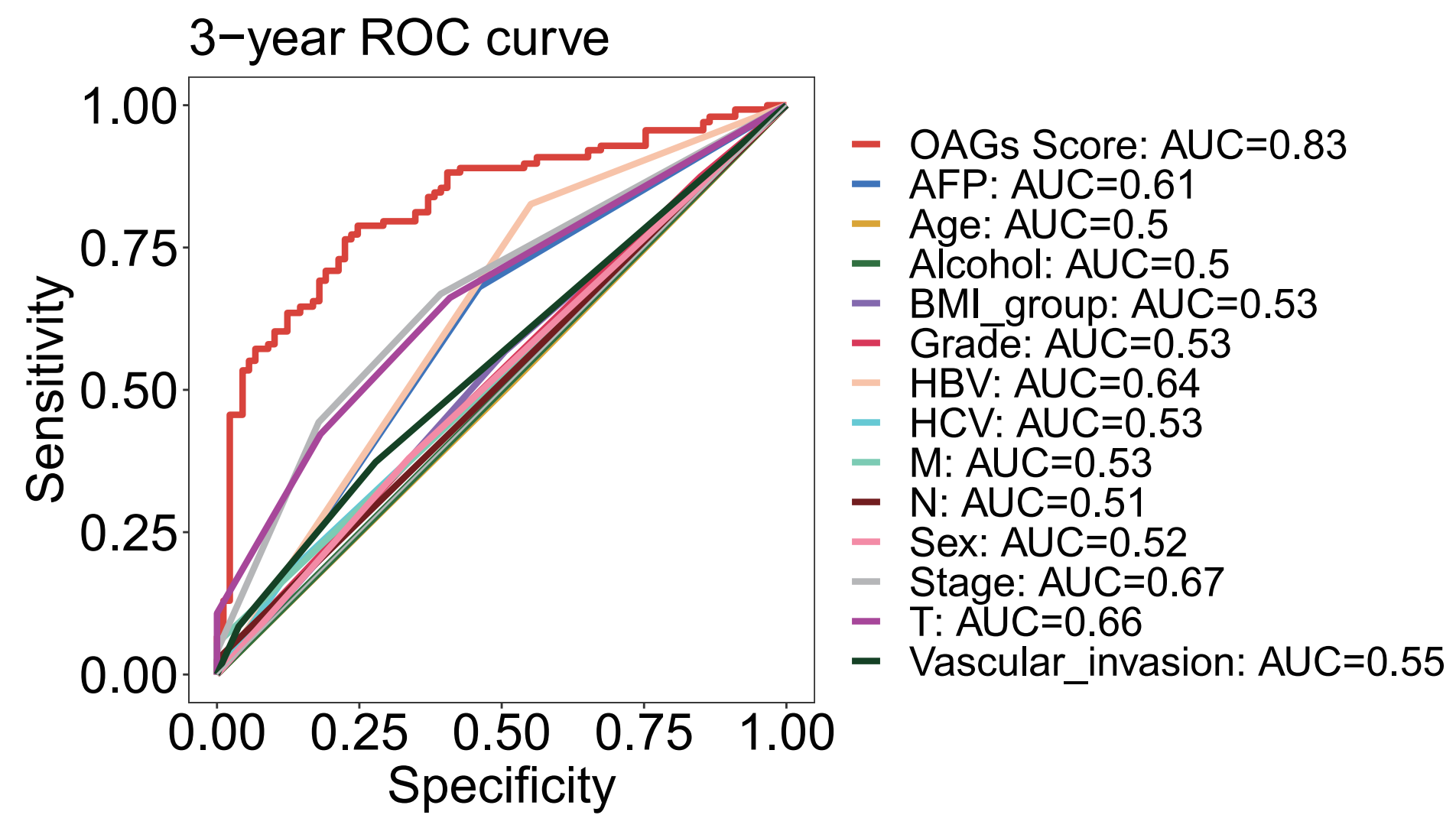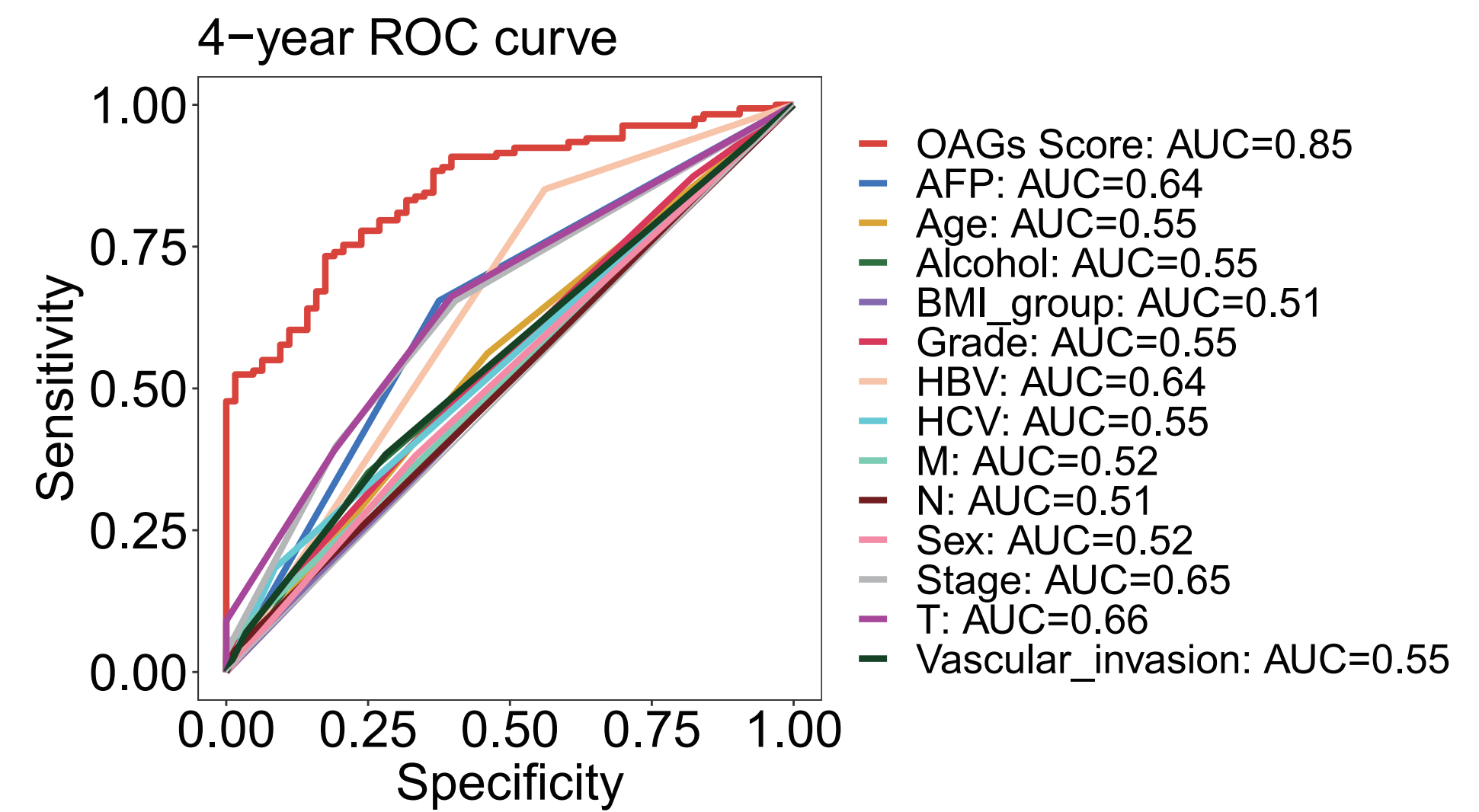

Supplement: Supplementary file 6 [file Image_6.pdf]

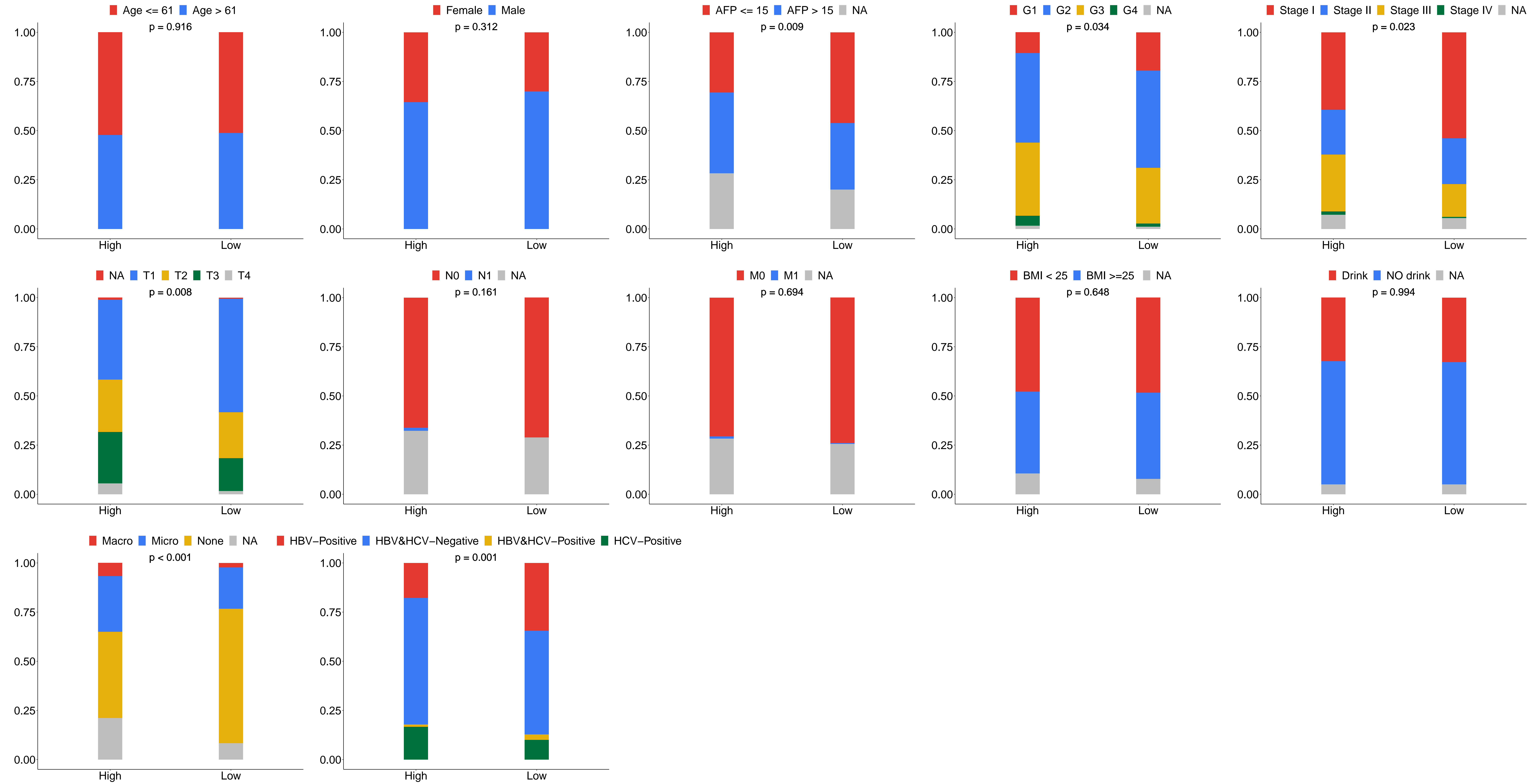

Supplement: Supplementary file 7 [file Image_7.pdf]

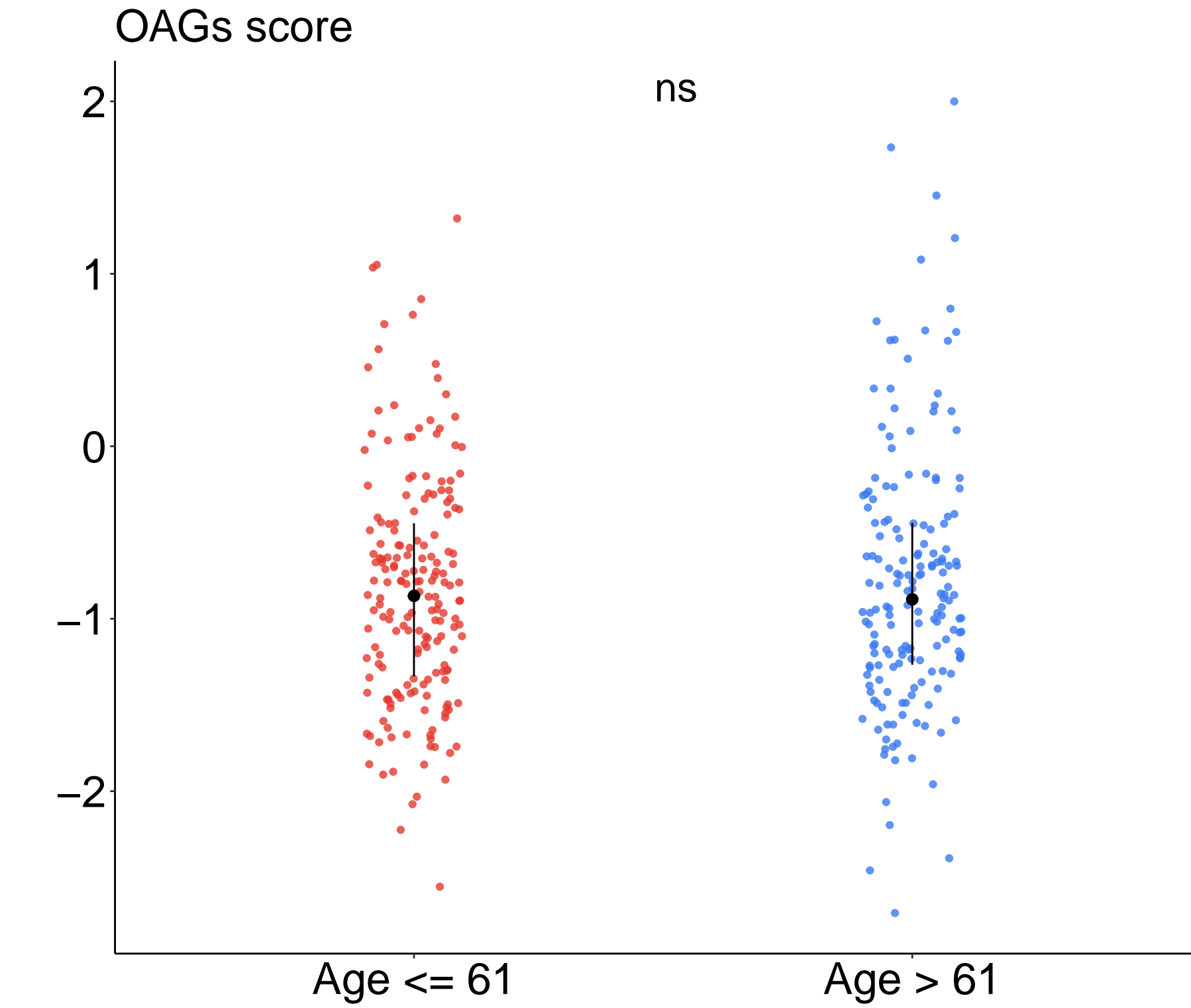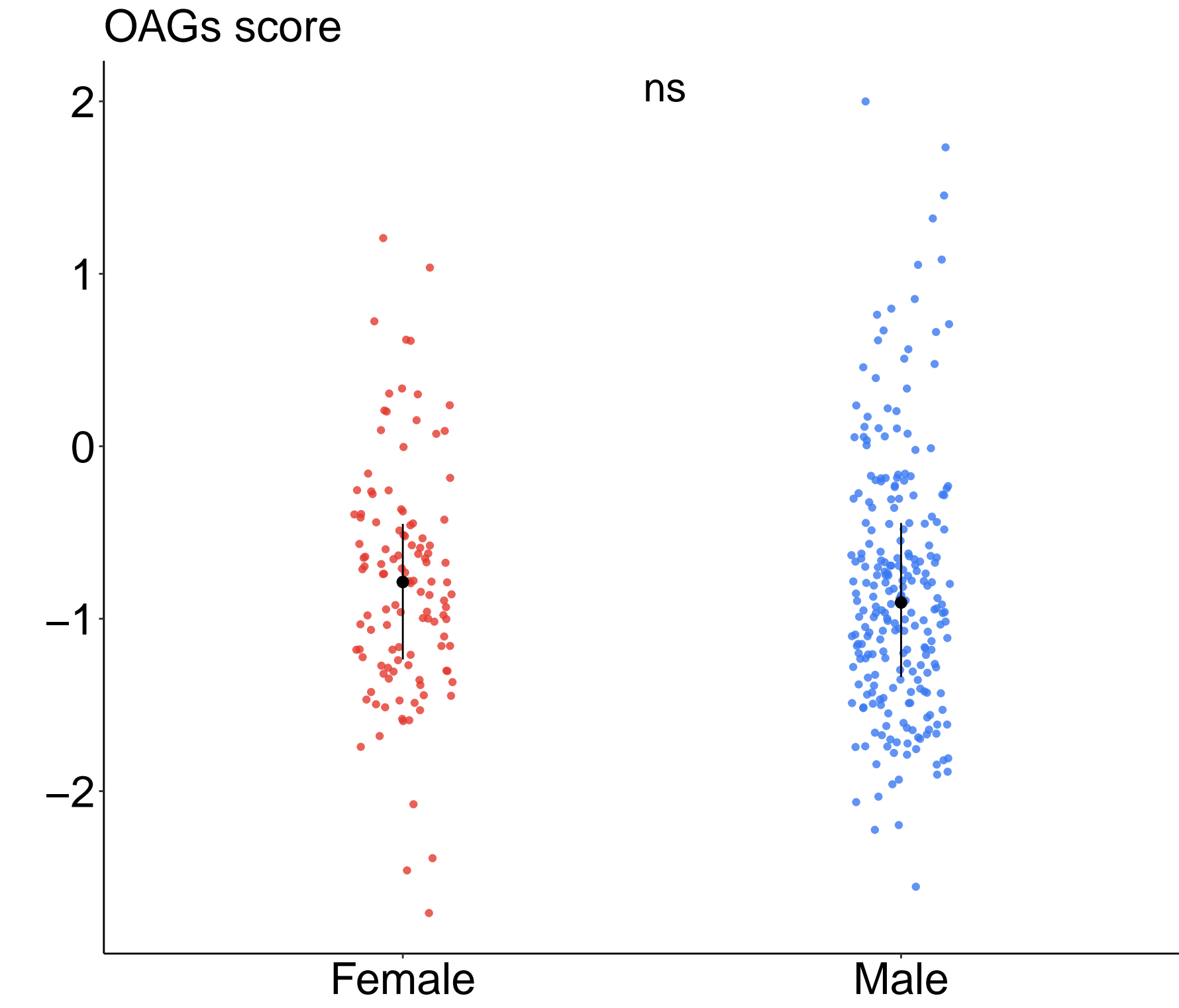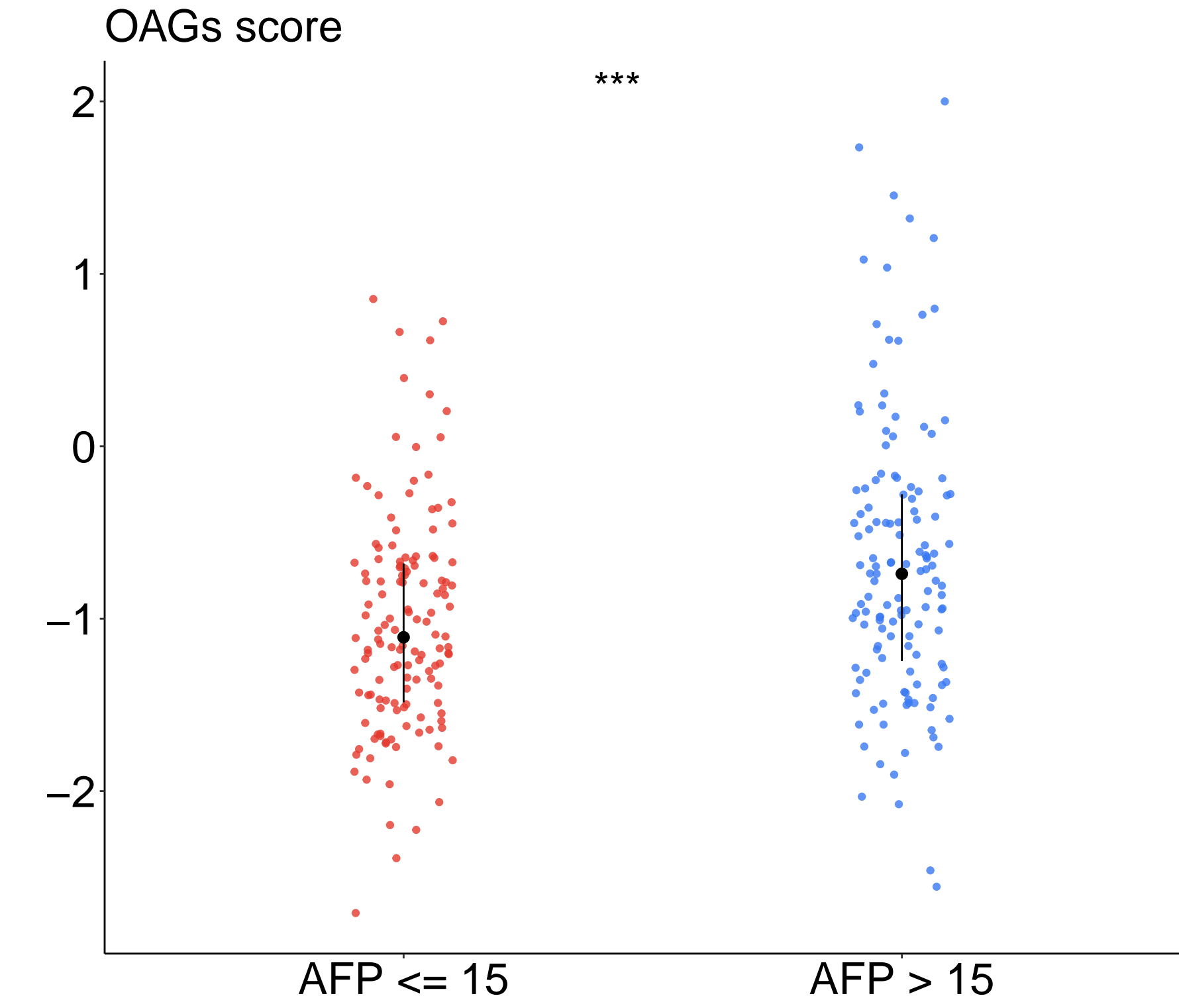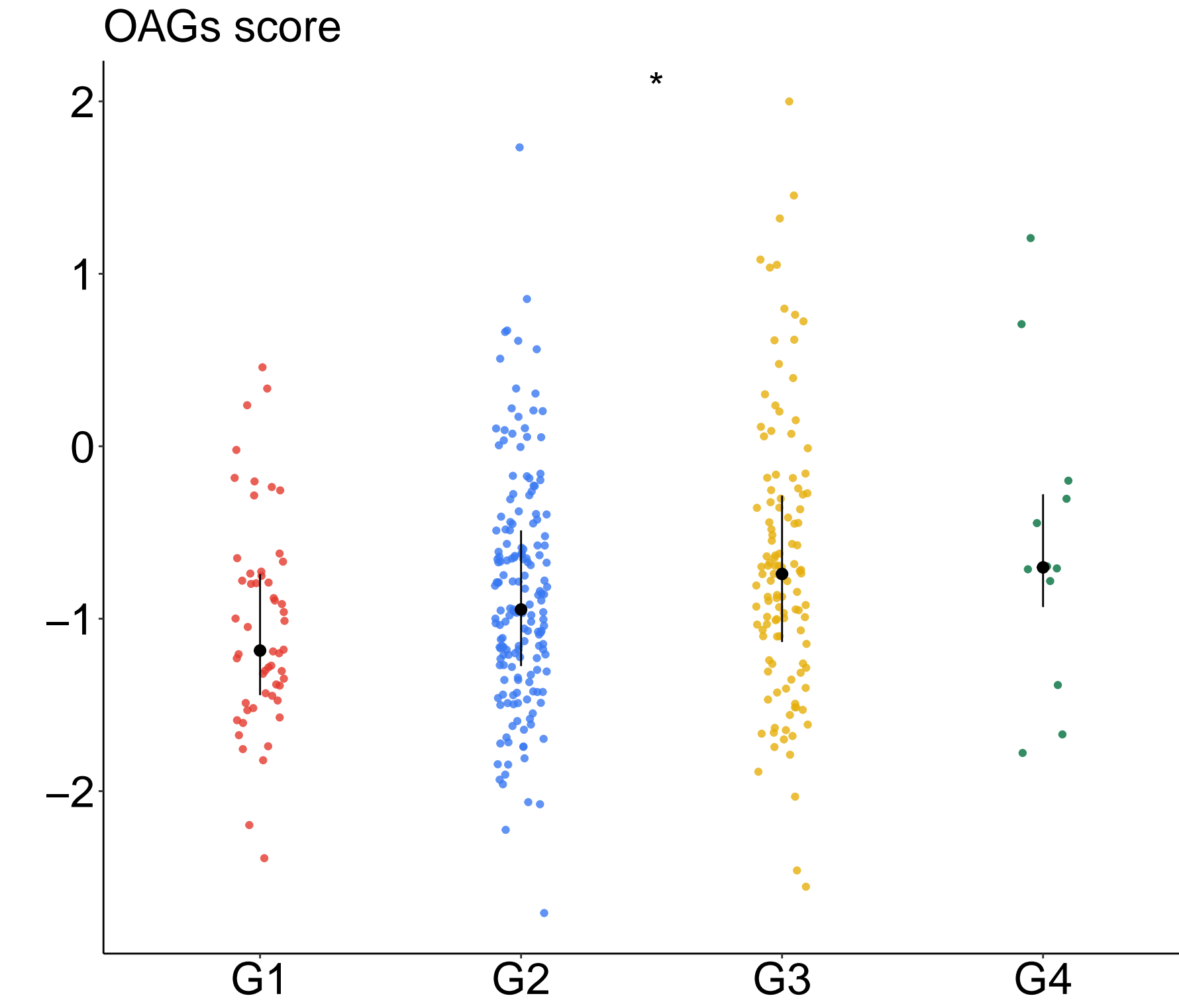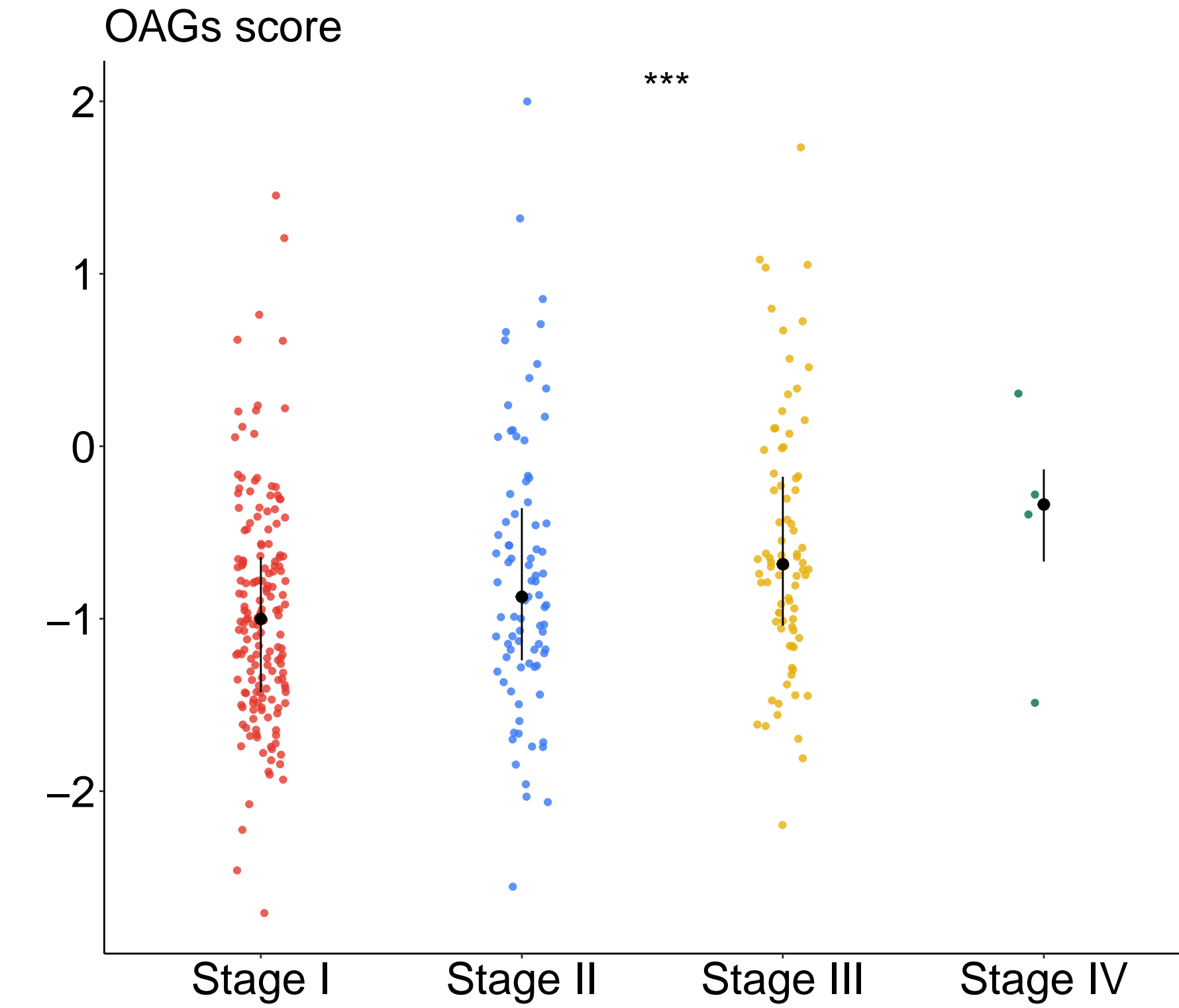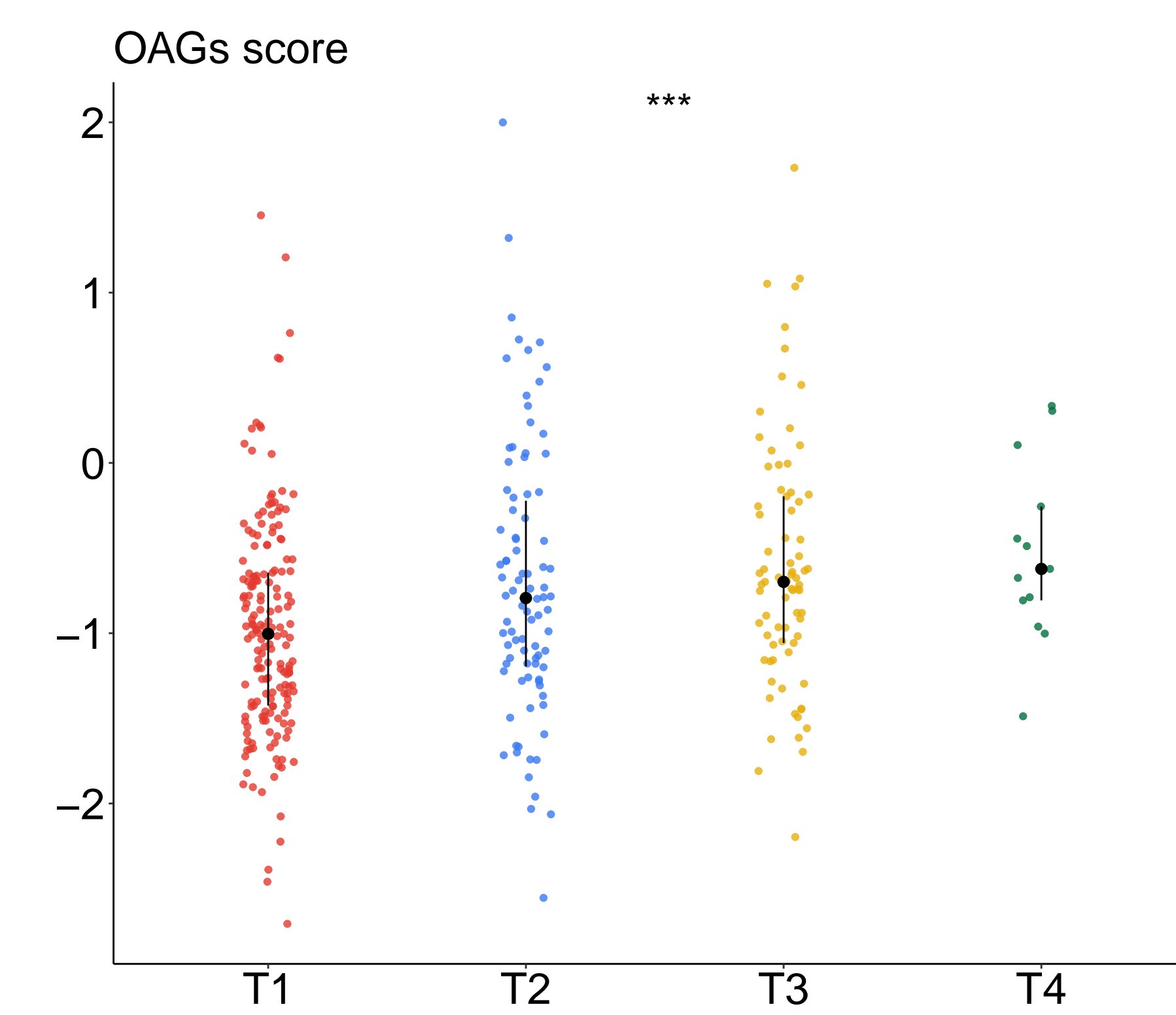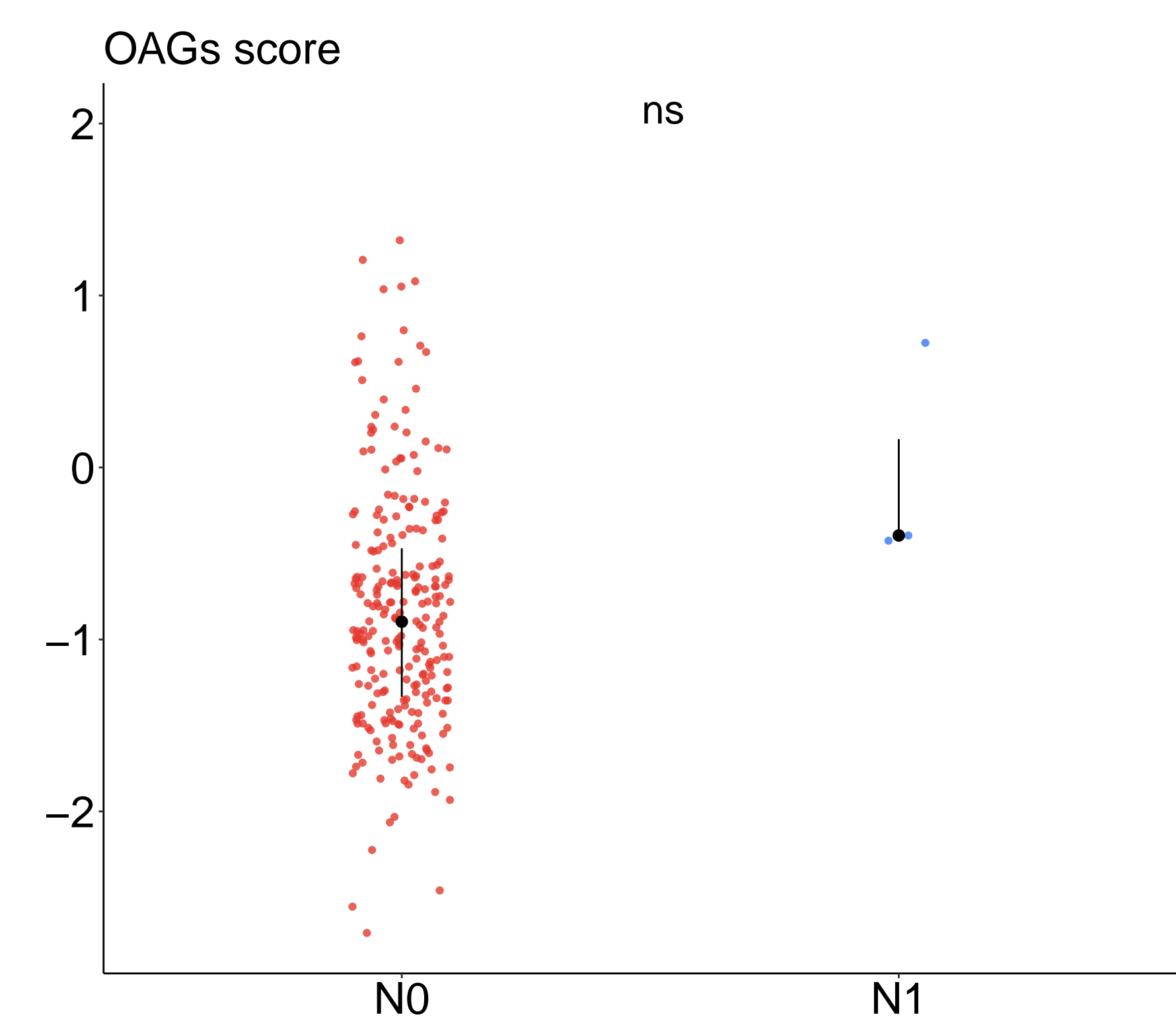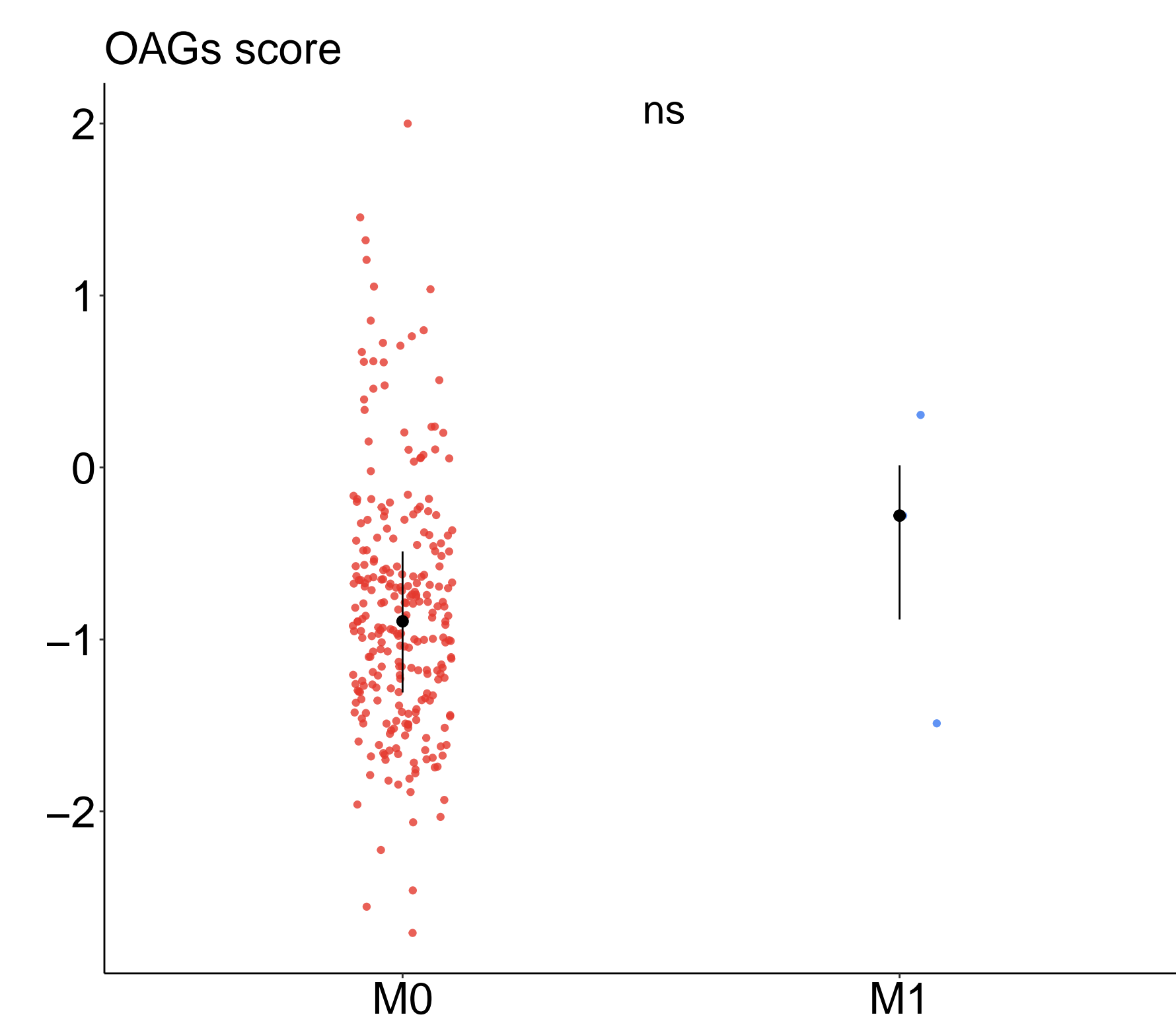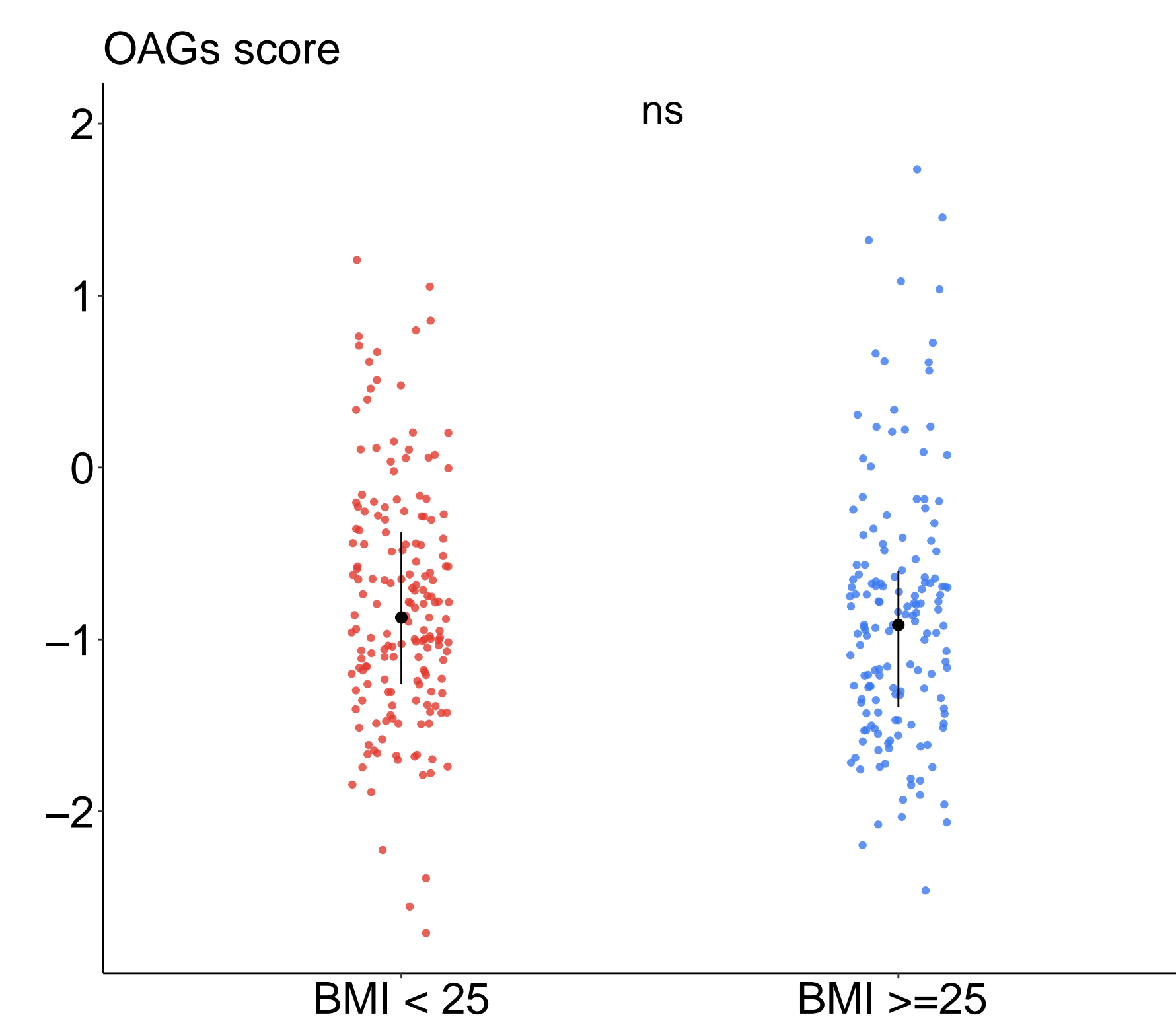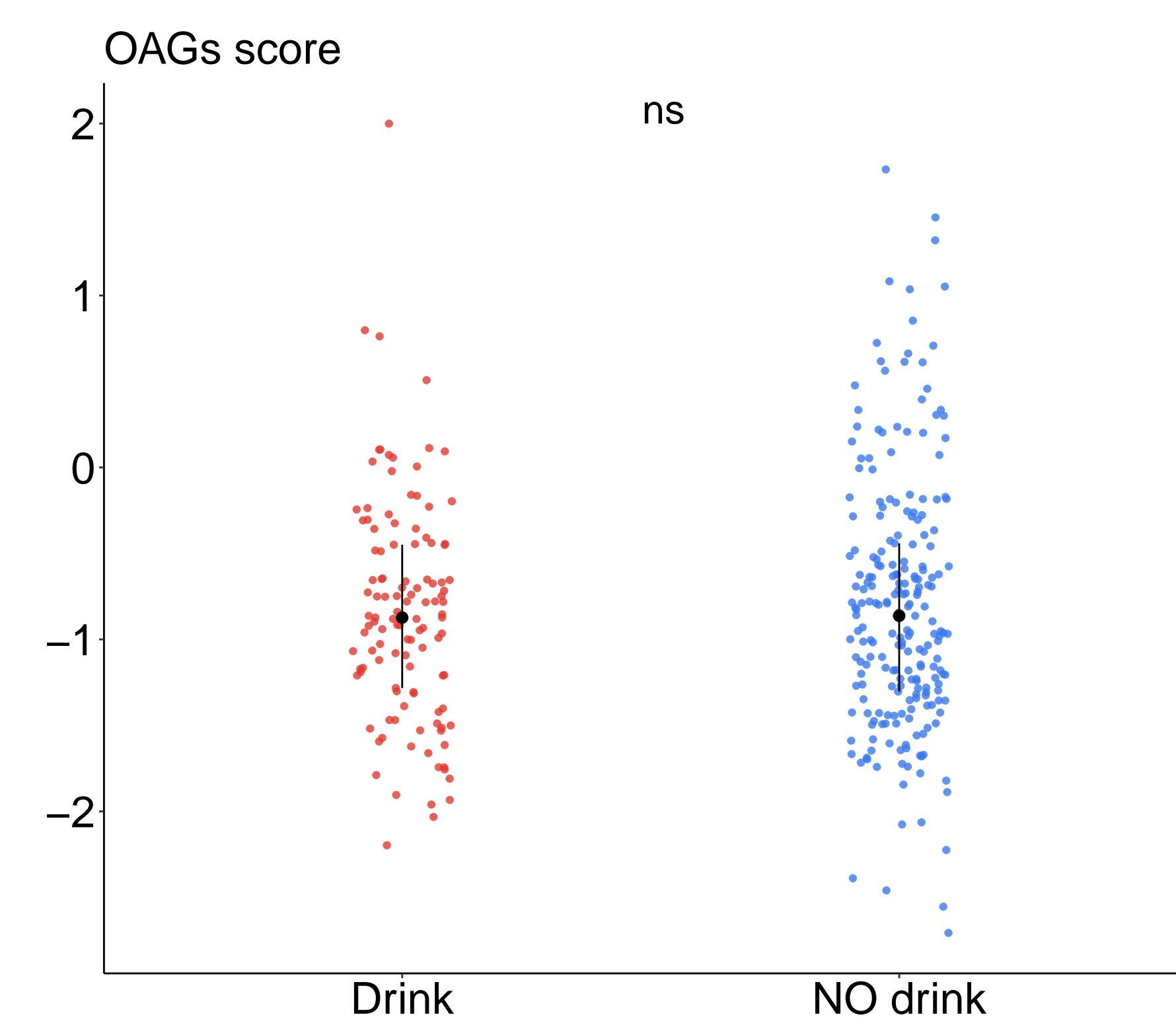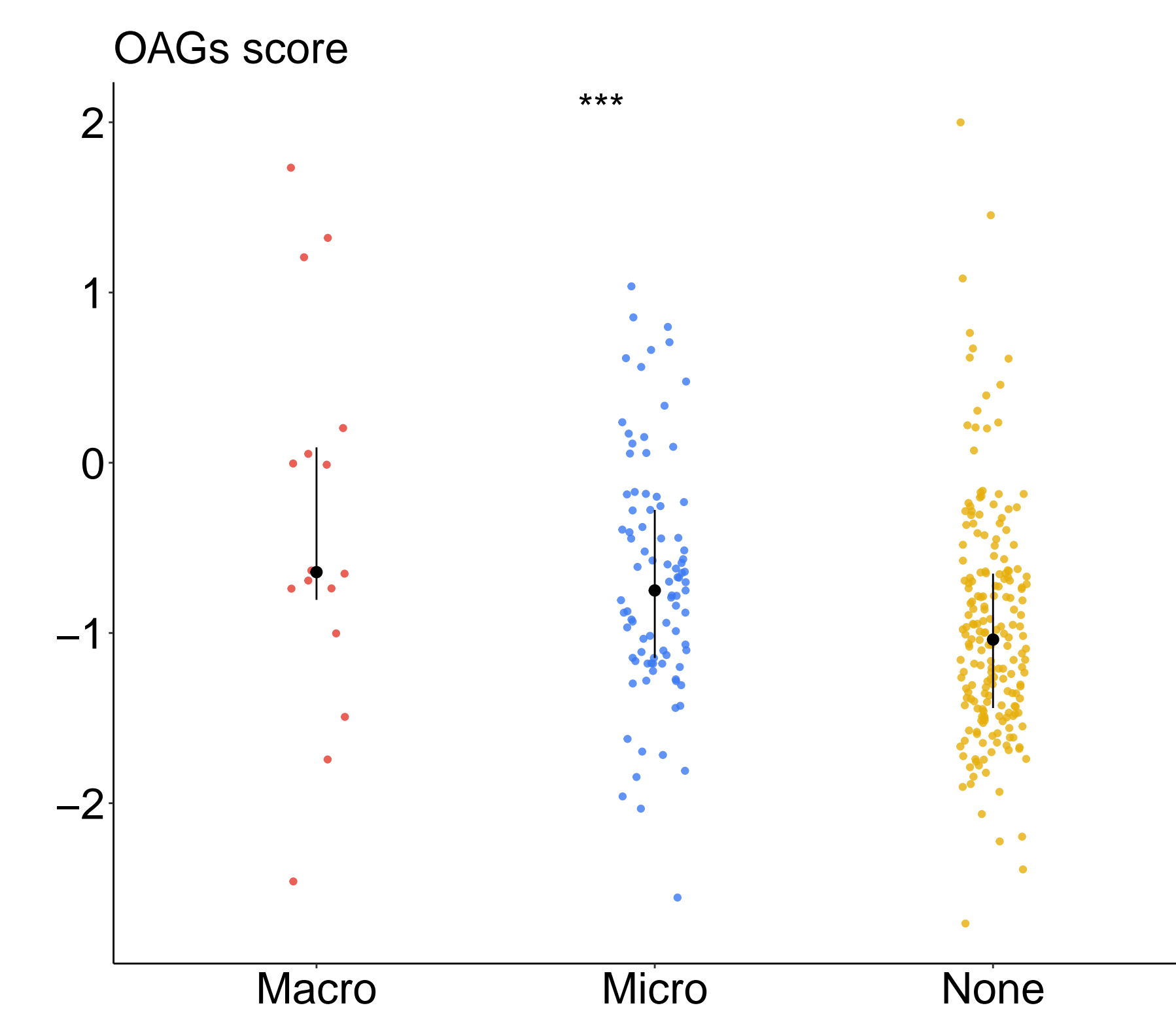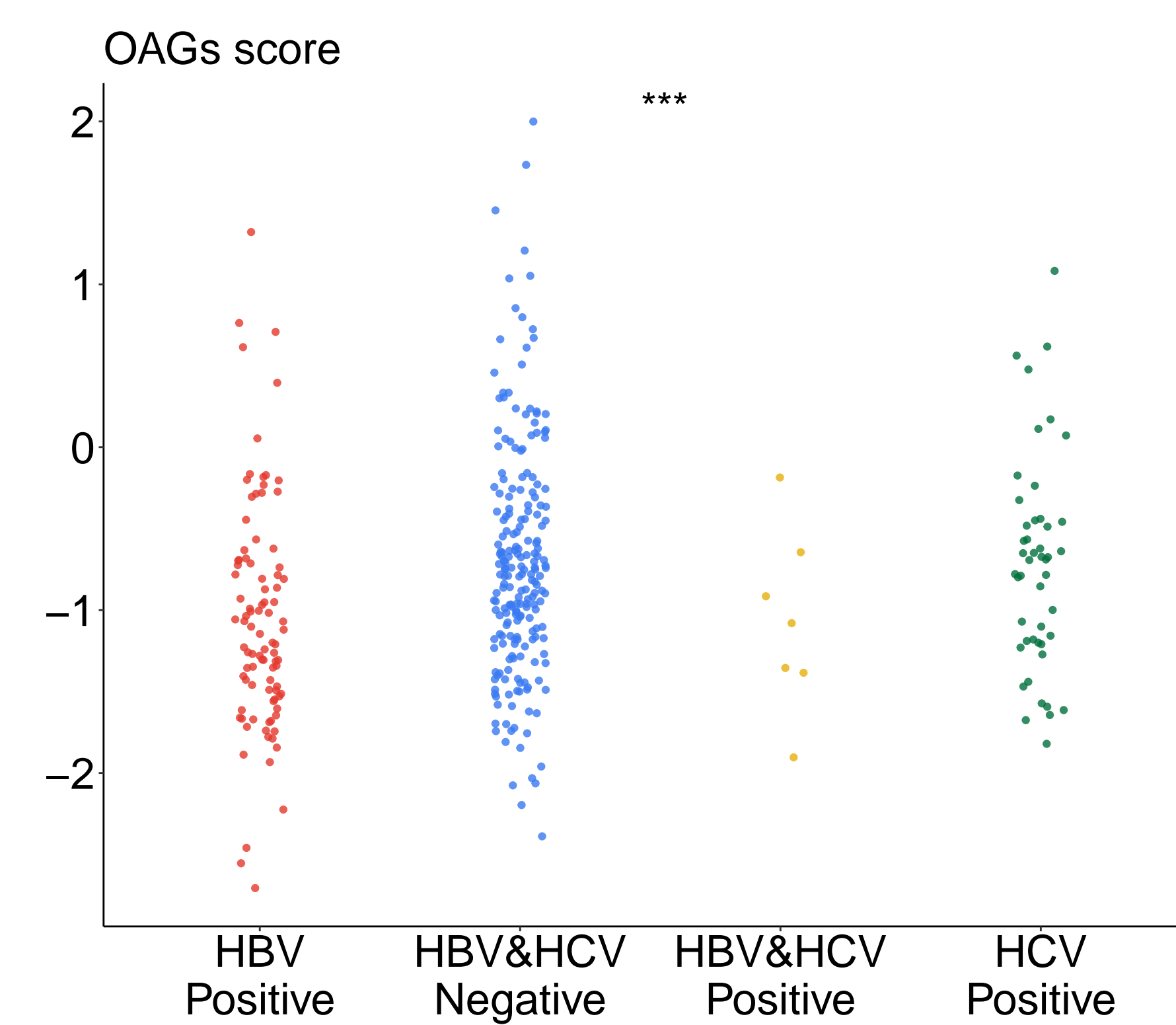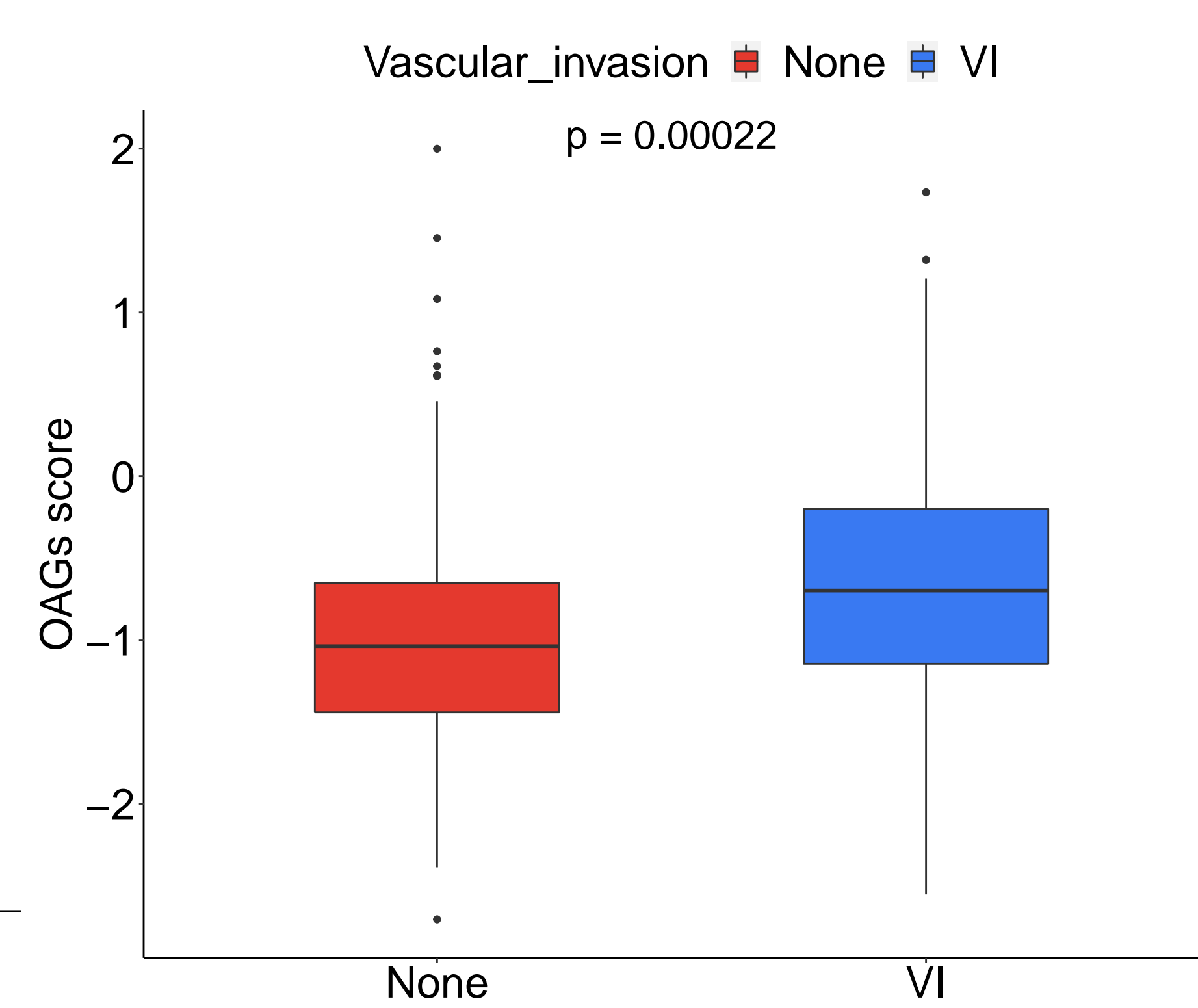

Supplement: Supplementary file 8 [file Image_8.pdf]

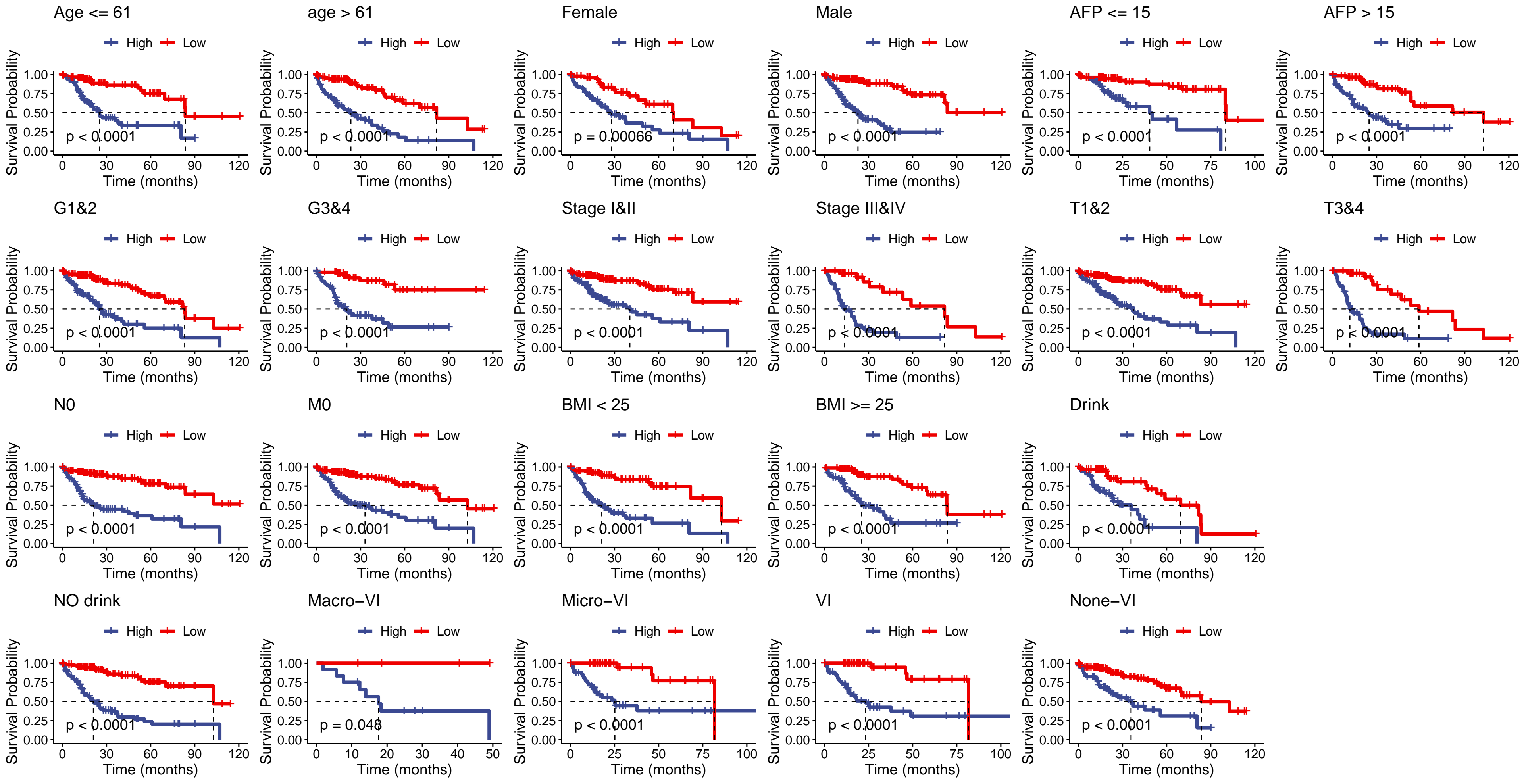

Supplement: Supplementary file 9 [file Image_9.pdf]
